# Heritability and genetic contribution analysis of structural-functional coupling in human brain

**DOI:** 10.1162/imag_a_00346

**Published:** 2024-10-30

**Authors:** Wei Dai, Zhengwu Zhang, Peihan Song, Heping Zhang, Yize Zhao

**Affiliations:** Department of Biostatistics, Yale University School of Public Health, New Haven, CT, United States; Department of Statistics, University of North Carolina at Chapel Hill, Chapel Hill, NC, United States

**Keywords:** structural connectivity, functional connectivity, structural connectivity-functional connectivity (SC-FC) coupling, heritability, GWAS

## Abstract

The flow of functional connectivity (FC) is thought to be supported by white matter structural connectivity (SC). While research on the correlations between SC and FC (SC-FC coupling) has progressed, the genetic implications of SC-FC coupling have not been thoroughly examined. Traditionally, SC-FC coupling investigations utilize predefined atlases. Here, we adopted an atlas-free SC-FC coupling built on the high-resolution white surface (the interface of white matter and gray matter) to uncover common genetic variations. Leveraging data from the Human Connectome Project, we demonstrated considerable heritability in areas within the early and intermediate visual cortex and across dorsal-attention, language, and somatomotor functional networks. We detected 334 genetic loci (spanning 234 cytogenetic bands) linked to SC-FC coupling (P < 1.26 × 10^−11^), notably in cingulo-opercular, somatomotor, and default mode networks. Using an external dataset from the Adolescent Brain Cognitive Development study, we confirmed 187 cytogenetic bands associated with SC-FC coupling across 22 brain regions (P < 1 × 10^-5^). Genetic correlation analyses revealed high genetic interrelatedness for SC-FC coupling in neighboring regions. Furthermore, it showed genetic correlations with a spectrum of complex traits, encompassing various neurological and psychiatric conditions. In essence, our study paves the way towards deciphering the genetic interplay between structural and functional connectivity of the brain.

## 1 Introduction

Understanding the complex human brain system requires characterizing its architectures across multiple areas—from gray matter (GM) to white matter (WM). GM contains the majority of neurons, enabling it to process information and release new information through axon signaling found in WM. Throughout the central nervous system, GM facilitates the control of movement, memory, and emotions, among other behaviors. In contrast, WM contains areas that primarily consist of myelinated axons. These axons connect various GM areas (the locations of nerve cell bodies) in the brain to each other and carry nerve impulses between neurons. With recent advances in neuroimaging techniques, we can construct different types of brain connectivity to summarize the relationships or communication between distinct brain regions through various imaging modalities in both GM and WM. Specifically, we can construct structural connectivity (SC) based on WM (Sporns, 2013) to measure the restriction of isotropic diffusive water movement using diffusion magnetic resonance imaging (dMRI); and functional connectivity (FC) within GM cortical regions (Sporns, 2013) to quantify statistical dependence of blood oxygen level-dependent (BOLD) time series measured by functional MRI (fMRI) (Ogawa et al., 1990). Rather than demarcating brain areas, both SC and FC focus on regional interactions and are crucial metrics for understanding the human brain (Sporns, 2013;Zimmermann et al., 2018).

Along with advances in studying SC and FC separately, there is growing interest in investigating the interrelationship between these two forms of connectivity, referred to as SC-FC coupling. The rationale behind this is that SC constrains FC, and FC, in turn, exerts effects on SC through mechanisms of plasticity (Zhang et al., 2011). Unlike FC (Cao et al., 2021), which focuses solely on functional connections between brain regions, SC-FC coupling captures the dynamic interplay between these functional connections and their supporting structural pathways. This approach provides a deeper layer of analysis, enabling us to explore how SC might influence or constrain FC and potentially identify genetic factors involved in coordinating brain networks—insights that FC alone may not fully capture. Studying SC-FC coupling, therefore, complements and extends the findings from FC studies, offering a more comprehensive understanding of the neurogenetic basis of psychiatric disorders. Although a few recent studies have already revealed SC-FC coupling alterations during normal development and in diseases (Chan et al., 2022;Skudlarski et al., 2010;Sun et al., 2017;van den Heuvel et al., 2013), the detailed mechanisms of how these variations are generated and related to behavioral outcomes remain undetermined, with inconsistent conclusions. For instance, some studies showed patients with schizophrenia experiencing decoupling between SC and FC in the posterior cingulate cortex (Skudlarski et al., 2010) while other studies have reported stronger SC-FC couplings in patients with schizophrenia (van den Heuvel et al., 2013). One possibility for the above disagreement on the coupling alteration in schizophrenia patients could be attributed to using different parcellations to construct SC and FC. To avoid arbitrarily choosing atlases,Cole et al. (2021)proposed the Surface-Based Connectivity Integration (SBCI), which used the white surface (the interface between white and gray matter) to create both SC and FC without the constraint of any pre-specified brain parcellation. They developed a new SC-FC coupling via inner product to assess the SC-FC correlation at each vertex on the cortical surface, demonstrating that this high-resolution SC-FC coupling had higher reproducibility and discriminability for sex detection using the Human Connectome Project Young Adult (HCP-YA) (Somerville et al., 2018). The SC-FC coupling was constructed in a continuous manner across the white surface, which will facilitate a more flexible and robust platform to study brain organization, and potentially better capture the links between structural and functional brain signals than those based on arbitrary atlases and parcellation (Messe, 2020).

A multitude of studies have been conducted to investigate the genetic impact on SC (Zhao, Li, Yang, et al., 2021;Zhao, Zhang, et al., 2021) and FC (Elliott et al., 2019;Ge et al., 2017;Miranda-Dominguez et al., 2018), respectively. A recent review summarized that brain functional organizations characterized by resting and task fMRI were moderately heritable, with heritability ranging from 20 to 60% (Foo et al., 2020). Heritability of WM tract-averaged fractional anisotropy ranged from 53 to 90% in a twin study of HCP-YA (Kochunov et al., 2015). In contrast, little attention has been given to investigate the genetic influence on the integrative SC-FC coupling. To the best of our knowledge, only one recent study (Gu et al., 2021) has focused on quantifying heritability for regional SC-FC coupling measures and discovered that they are highly heritable, especially in the subcortical, cerebellum/brainstem, and visual networks. This highlights the opportunity to uncover genetic polymorphisms supporting SC-FC coupling by dissecting the genetic bases associated with connectivity coupling alterations. Such coupling-related genetic underpinnings have not yet been explored in any previous studies, nor have their shared genetic influences with other complex traits and clinical consequences.

To lay the groundwork for uncovering the genetic bases for SC and FC coupling, we performed a series of genetic association analyses for the latest SC-FC coupling under the framework ofCole et al. (2021)using HCP-YA. Unlike existing coupling measures, including those inGu et al. (2021)which were constructed by calculating correlation between SC and FC adjacency matrices derived from a predefined atlas, the coupling measures used in this study were built from the white surface via inner-product consisting of 3,726 vertices in a continuous paradigm after masking out the corpus callosum region. SC-FC coupling constructed in this manner represents how each brain region (vertex) is connected, in terms of structure and function, to all other regions (vertices), summarizing multimodal connectivity measures into a single measure. We thoroughly explored the genetic architecture of SC-FC coupling and further investigated the shared genetic architecture between the novel SC-FC coupling and complex traits (e.g., brain-related disorders) to reveal its potential as an innovative biomarker for understanding how genetics influence brain structures, circuits, and, in turn, cognitive behavior.

## 2 Methods

### 2.1 Datasets

The data for this study were mainly from a large population of young and healthy adults in the HCP Young Adult (HCP-YA) project downloaded from the ConnectomeDB website (Somerville et al., 2018). Our data usage was approved by HCP and complies with all relevant ethical regulations for work with human participants. Specifically, we use WU-Minn HCP minimally processed S1200 release, which includes preprocessed T1-weighted (T1w), dMRI images and unprocessed resting-state fMRI images, demographics, behavioral, and cognitive scores for a population of 1,113 young healthy adults. The resting-state fMRI data were collected in two sessions and each session consisted of two resting-state acquisitions of approximately 15 minutes, which was used for test-retest reliability of heritability estimation. These subjects (age, 29.21 ± 3.47 years; female, 55.84%) come from 457 families and comprise 149 MZ twin pairs and 94 DZ twin pairs. Further details about the recruitment process, imaging data acquisition, inclusion, and exclusion criteria were detailed in (Smith et al., 2013;Somerville et al., 2018;van den Heuvel et al., 2013;Van Essen et al., 2012).

The genotyping data for 1,114 subjects were released by HCP and available through the dbGAP repository with study session: phs001364.v1.p1. All subjects were genotyped using the Illumina Multi-Ethnic Global Array (MEGA) SNP-array that included chip-specific content from PsychChip and ImmunoChip and provides extended coverage of European, East Asian, and South Asian populations. We used 1,064,964 SNPs that satisfied the following quality controls with both imaging and genetics data available (n = 899): (1) excluding subjects with more than 10% missing genotypes; (2) excluding variants with minor allele frequency (MAF) less than 0.01; (3) excluding variants with missing genotype rate larger than 10%; and (4) excluding variants that failed the Hardy-Weinberg test at 1 × 10^−7^level.

For validation, we include 1,546 subjects from Adolescent Brain Cognitive Development (ABCD) study, with similar demographic features to samples in HCP dataset. These subjects (age, 9.95 ± 0.71 years; female, 50.45%) include 878 white, 297 Hispanic, 179 Black, 29 Asian, and 163 other ethnicity/race (Supplemental Table S10). The genotype array includes 332,057 SNPs and can be accessed by submitting a request athttps://nda.nih.gov/abcd/request-access.html. The imaging phenotype was obtained through NIMH Data Archive (NDA) and processed with the same pipeline as HCP.

For the heritability and GWAS analyses, we analyzed 899 subjects (age, 28.69 ± 3.69 years; 419 males, 480 females) from 419 different families, including 140 MZ twin pairs, 85 DZ twin pairs, 280 full siblings, and 204 singletons (single-birth individuals without siblings). For sensitivity analysis on the white and non-Hispanic only subjects, we included 625 subjects (age, 29.01 ± 3.53 years; 329 males, 296 females) from 288 different families, including 112 MZ twin pairs, 72 DZ twin pairs, 145 full siblings, and 157 singletons. More details on the inclusion of subjects and their demographic information can be found inSupplemental Figure S12andSupplemental Table S7.

### 2.2 Construction of SC-FC coupling

We first downloaded the minimally preprocessed diffusion MRI data from ConnectomeDB. The diffusion images were processed with steps including b0 intensity normalization, susceptibility-induced distortion correction, and motion correction and eddy current correction. Detailed steps can be found inGlasser et al. (2013). The anatomical T1w image processing included registration via ANTs and surface reconstruction using the recon_all tool available in Freesurfer (http://freesurfer.net/). We also downloaded the processed resting-state fMRI in the CIFTI format. More details about the imaging preparation and preprocessing can be found inGlasser et al. (2013)andCole et al. (2021).

SC-FC coupling was constructed following the surface-based connectivity integration (SBCI) framework inCole et al. (2021). First, the SC and FC were both constructed on the white surface, the interface between cortical GM and WM. Since diffusion signals were mostly in the WM regions and functional signals were more present in the GM regions, the white surface was more suitable to study the integration of SC and FC. For any two points on the white surface, we obtained a continuous FC through the signal interpolation method and smoothed the sparse SC using kernel-based method. Since we considered SC and FC as functional data, we used the widely accepted inner product to quantify similarity between SC and FC as below. When SC and FC were very similar, we got a value close to 1, when they were orthonormal to each other we got 0, and when they were complete opposite to each other, we got -1. Specifically, the SC-FC coupling at each vertex$x_{0}$on the white surface was calculated using a normalized inner project of two functions$f_{1}(y)=\ell_{SC}(x_{0},y)$and$f_{2}(y)=\ell_{FC}(x_{0},y)$:

$$SFC(x_{0})=\langle \frac{f_{1}^{E}}{\left|\left|f_{1}^{E}\right|\right|},\frac{f_{2}^{E}}{\left|\left|f_{2}^{E}\right|\right|}\rangle =\langle \frac{\int_{s\;\in \;E}\ell_{SC}(x_{0},s)\ell_{FC}(x_{0},s)ds}{\sqrt{\int_{s\;\in \;\text{E}}\ell_{SC}^{2}(x_{0},s)ds\int_{s\;\in \;\text{E}}\ell_{FC}^{2}(x_{0},s)ds}}\rangle ,$$

where$\ell_{SC}(x,y)$and$\ell_{FC}(x,y)$denotes the continuous SC and FC for a particular subject between two verticesxandy;Eis a local brain region containing$x_{0}$;〈,〉is the inner product operator; and$SFC(x_{0})$represents SC-FC coupling at$x_{0}$. We used seven functional networks (visual, somatomotor, dorsal attention, ventral attention, limbic, frontoparietal, and default mode networks) fromYeo et al. (2011)to define the$E^{\prime }s$to better localize the FC (e.g., FC data within each local regionEare more consistent). We used log-transformation of SC to account for the non-normality of the entries in the SC and Pearson correlation-based FC for the calculation. The SC-FC coupling calculated in this way was a continuous function measuring the similarity/consistency between SC and FC at different locations on the white surface. In practice, the dense vertices on the mesh surfaces from Freesurfer caused computational challenges. In our implementation of SBCI, we downsampled each white surface mesh (left and right) from over 120,000 vertices to around 2,100 without masking out the corpus callosum region. The result of this calculation led to a vector of length 3,726 that represents the SC-FC coupling strength for each of the 3,726 vertices across the brain after masking out the corpus callosum region for each individual. We refer the readers to the original paper (Cole et al., 2021) for more details. The SC-FC coupling characterizes how SC between a given location and all other locations in the brain is related to FC at that location and all other locations.

We also performed several ancillary analyses to verify the robustness of our SC–FC coupling results to the choices in method of calculating SC-FC coupling where we also constructed the SC–FC coupling by calculating the Pearson’s and Spearman-rank correlation between$\ell_{SC}(x_{0},s)$and$\ell_{FC}(x_{0},s)$fors∈E.

### 2.3 Heritability analysis

SNP heritability for each SC-FC coupling, SC and FC node strength was estimated by GCTA-GREML (Yang et al., 2011) with a linear mixed effect (LME) model using all autosomal SNPs. Though original GCTA approaches were specifically developed to estimate heritability using unrelated individuals (Yang et al., 2010), the heritability estimates by GCTA were shown to be accurate for related samples (Zaitlen et al., 2013). In a recent study, it was used to estimate heritability for 60 regional and 1.3 × 10^5^voxel-wise traits in 1,206 twin and sibling participants from the HCP and 37,432 participants from the UK Biobank (UKBB) (Gao et al., 2021) and demonstrated the agreement of heritability from two datasets with Pearson’s correlation ranging from 0.63 to 0.76. Their findings can contribute to the conclusion that the heritability estimation of GCTA is reliable for both related and unrelated samples. Specifically, we applied the following model for estimating h^2^:y=μ1+Zu+e, where**e **$∼\text{N}\left(0,\text{I}\sigma_{e}^{2}\right)$and**u**$∼N\left(0,\;\;\text{K}\sigma_{g}^{2}\right)$**.**The matrix**K**is the genetic similarity matrix estimated as defined in (Yang et al.). Thus, the covariance matrix ofyis$\text{cov}(\text{y})=\text{K}h^{2}\;+\text{I}(1-h^{2})$, where$h^{2}\;=\;\;\sigma_{g}^{2}/\left(\sigma_{g}^{2}+\sigma_{e}^{2}\right)$is the heritability, that is, the proportion of phenotypic variance explained by the causal variants altogether.

We estimated the heritability for SC-FC coupling, SC and FC node strength adjusting the effects of age (at imaging), age-squared, sex, age-sex interaction, age-squared-sex interaction, handedness, total brain volume, and the top 10 genetic principal components (PCs). Additionally, because there may be differences in genetic similarity patterns across race/ethnicity, we recalculated heritability of SC-FC coupling using a homogeneous subset of white, non-Hispanic individuals (n = 625).

To make a fair comparison with results inGu et al. (2021), we used the same approach, a linear mixed-effects model for repeated measures, asGu et al. (2021), to estimate heritability using two resting-state fMRI scans. This LME model can disentangle inter- versus intra-subject variation. Unlike in the previous setting where the genetic similarity matrix was estimated based on genotype, the genetic similarity matrix in this approach was derived from the pedigree information where 1 for monozygotic twins, 1/2 for dizygotic twins, and full siblings and 0 for unrelated individuals. We adjusted the same set of covariates in the heritability estimation.

Given the moderate sample size and related individuals might potentially interfere heritability estimation, we performed sensitivity analyses with simulated data to evaluate their effects. We used the genotype data in our analyses for simulation and generated a single phenotype with known heritability${\text{h}}_{\text{true}}^{2}$= 0.4 fromy∼N(μ,Σ)with$\Sigma =\;\;\text{K}{\text{h}}_{\text{true}}^{2}\;\;+\text{I}\left(1-{\text{h}}_{\text{true}}^{2}\right)$andμ=Xβ. The matrix**K**is the genetic similarity matrix estimated following the method (Yang et al., 2010). Theμrepresented the total genetic effect from causal SNPs in the matrix**X**. We randomly selected 500 SNPs as causal SNPs with effect sizesβ∼N (-0.5, 0.5). This procedure was conducted on different sub-populations generated under the following two ways: (1) randomly selected a certain proportion of the samples ranging from 50% to 90% from the whole dataset. (2) adjusted the relatedness level by first only including unrelated samples (one from each family) and gradually adding 10–90% related samples. For both situations, after synthesizing the phenotype data, we re-estimated the heritability h^2^and calculated the squared difference${\left({\hat{h}}^{2}-h_{true}^{2}\right)}^{2}$. This entire process, encompassing data generation and evaluations, was repeated 4,000 times, with the average squared difference and standard error of h^2^estimation reported across all iterations.

### 2.4 Genome-wide association (GWA) analysis

We adopted the linear mixed model (LME) in GCTA-fastGWA (Povysil et al., 2019) to account for genetic relatedness in the dataset for GWA, while adjusting for the same set of covariates. A significance threshold of 1.26 × 10^−11^was established (i.e., a stringent threshold determined by 0.05 / 1,064,964×3,726). For validation purpose, we include 1,546 subjects from ABCD study and set the significance level to be 1 × 10^-5^. The loose threshold enabled us to include as many signals as possible aiming to provide an early-stage guidance and sense on genetic basis of coupling.

To aid interpretation of GWA results, SC-FC coupling at each vertex across the brain was mapped to 360 regions under the Glasser360 atlas (Supplemental Table S8) (Jiang et al., 2021). Specifically, we summed over the number of significant SNPs for locations within each atlas and denoted it as the number of significant SNPs for that specific atlas. The 360 Glasser regions were further formed into 12 functional networks (Somerville et al., 2018): Somatomotor, Default, Frontoparietal, Cingulo-Opercular, Language, Auditory, Dorsal-Attention, Orbito-Affective, Visual I, Visual II, Posterior-Multimodal, and Ventral-Multimodal; or grouped into 22 cortices (Ji et al., 2019) (Supplemental Table S1) that belonged to 6 anatomical networks as suggested inGlasser et al. (2016): early and intermediate visual cortex (Primary Visual, Early Visual, Dorsal Stream Visual, Ventral Stream Visual, MT+ Complex and Neighboring Visual Areas), sensorimotor cortex (Somatosensory and Motor, Paracentral Lobular and Mid Cingulate, Premotor, Posterior Opercular), auditory cortex (Early Auditory, Auditory Association, Insular and Frontal Opercular), temporal cortex (Medial Temporal, Lateral Temporal), posterior cortex (Temporo-Parieto-Occipital Junction, Superior Parietal, Inferior Parietal, Posterior Cingulate), and anterior cortex (Anterior Cingulate and Medial Prefrontal, Orbital and Polar Frontal, Inferior Frontal, Dorsolateral Prefrontal).

We further used the Glasser360 atlas and the associated 12 functional and 5 anatomical networks to evaluate the enrichment of identified associations in various brain through the chi-squared test. Specifically, for each network, we constructed a contingency table that displayed the number of associations that were significant in the network, significant outside the network, insignificant in the network, and insignificant outside the network. Then, Pearson’s chi-squared test (Tamussino et al., 2001) was performed as an enrichment analysis. Similarly, we also conducted the enrichment analysis of locus-specific genetic effects. Specifically, we constructed a contingency table that displayed the number of significant associations that were in a cytoband genomic region and a brain network, in a cytoband genomic region but outside a brain network, not in a cytoband genomic region but in a brain network, and neither in a cytoband genomic region nor a brain network. The same Pearson’s chi-squared test was conducted to assess the significance.

### 2.5 The shared loci and genetic correlation

The genomic loci associated with the SC-FC coupling traits were defined using FUMA (Watanabe et al., 2017) (version 1.3.5e). To define the linkage disequilibrium (LD) boundaries, FUMA identified independent significant variants, which were defined as variants with a P-value smaller than the predefined threshold and were independent of other significant variants (LD r^2^< 0.6). FUMA then constructed LD blocks for these independent significant variants by tagging all variants in LD (r^2^≥ 0.6) with at least one independent significant variant and had an MAF ≥ 0.0005. These variants included those from the 1,000 Genomes reference panel that might not have been included in the GWAS. Moreover, within these significant variants, independent lead variants were identified as those that were independent of each other (LD r^2^< 0.1). If LD blocks of independent significant variants were close (<250 kb based on the closest boundary variants of LD blocks), they were merged into a single genomic locus. Thus, each genomic locus could contain multiple significant variants and lead variants. We then found the corresponding cytogenetic region for each significant variant using ANNOVAR (K. Wang et al., 2010). Independent significant variants and all the variants in the same cytogenetic region were searched on the NHGRI-EBI GWAS catalog (Buniello et al., 2019) (version 2022-01-12) to look for previously reported associations (P < 5 × 10^−6^) with any traits.

Pairwise genetic correlation between SC-FC coupling traits was estimated using GCTA-GRMEL analysis (Yang et al., 2011), while LDSC software (version 1.0.1) (B. Bulik-Sullivan et al., 2015;B. K. Bulik-Sullivan et al., 2015) was used to estimate and test the pairwise genetic correlation between SC-FC coupling and complex traits. For LDSC, we overlapped HCP genotype data with 1,000 Genomes European data and calculated LD scores with LDSC. The summary statistics for SC-FC coupling traits were from GWAS, and the resources of other summary statistics were provided inSupplemental Table S5.

## 3 Results

### 3.1 SC-FC coupling biomarkers are heritable and different from the heritability of SC or FC

**Fig. 1.**
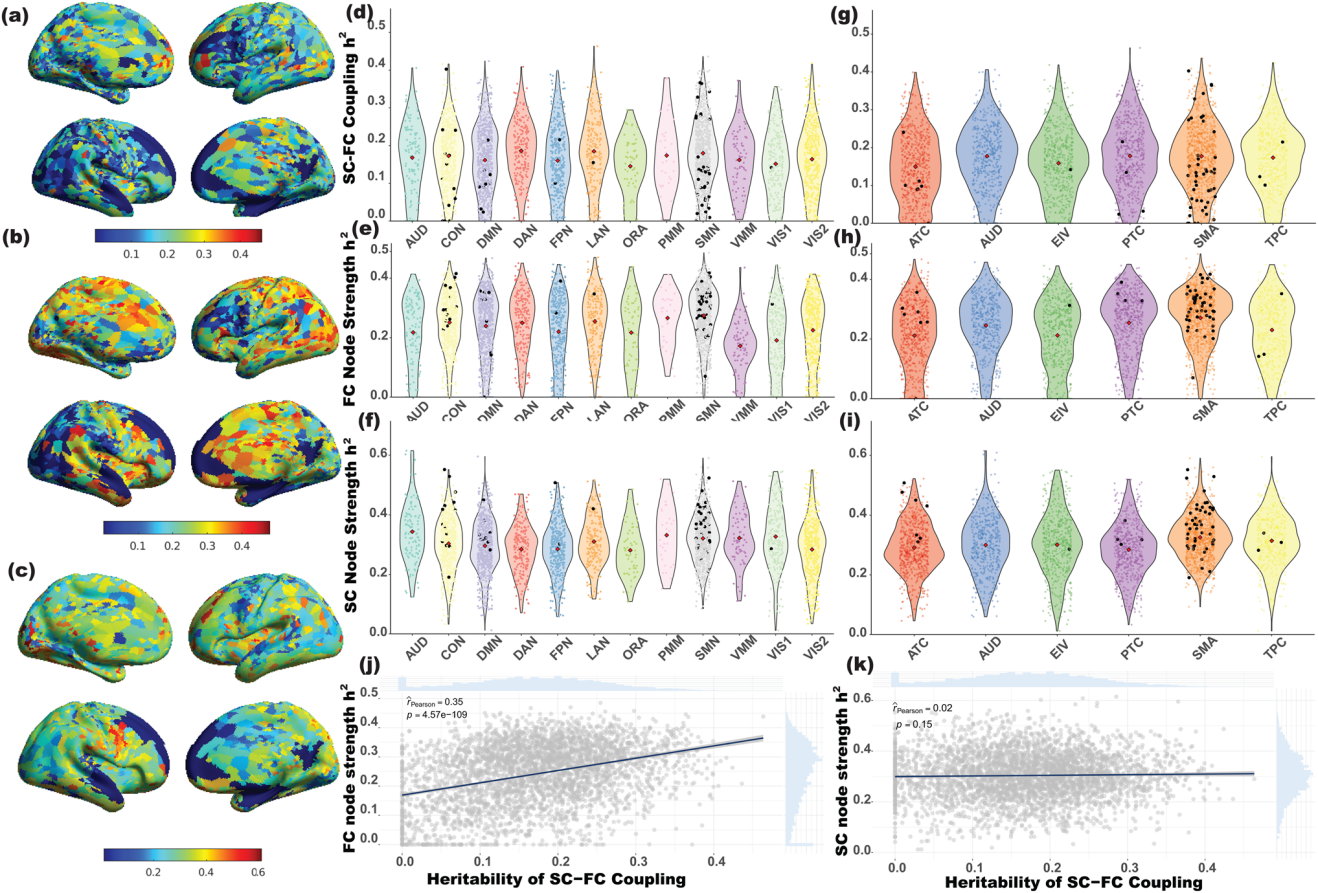
SC-FC coupling heritability estimates. (a), (b), and (c) SNP heritability for SC-FC coupling, FC node degree, and SC node degree. The color bar indicates the heritability value (0-1). (d), (e), and (f) Distributions of heritability estimates across SC-FC coupling, FC node degree, and SC node degree within functionally defined networks (Glasser et al., 2016): AUD — auditory; CON — cingulo-opecular; DMN — default mode; DAN — dorsal attention; FPN — frontoparietal; LAN — language; ORA — orbito-affective; PMM — posterior multimodal; SMN — somatomotor; VMM — ventral multimodal; VIS1 — primary visual; VIS2 — secondary visual. (g), (h), and (i) Distributions of heritability estimates across SC-FC coupling, FC node degree and SC node degree within anatomically defined networks (Glasser et al., 2016): ATC — anterior cortex; AUD — auditory regions; EIV — early and intermediate visual cortex; PTC — posterior cortex; SMA — sensorimotor areas; TPC — temporal cortex. SC-FC coupling traits with significant SNPs in genome-wide association results were represented as black dots. The average heritability within each network was represented by red diamond point. (j) and (k) Heritability estimates of SC–FC coupling were moderately positively correlated with the heritability of FC node strength (Pearson’s r = 0.35, P < 1e-5) and almost not correlated with heritability of SC node strength (Pearson’s r = 0.02, P = 0.15). The translucent bands around the regression line represent 95% confidence interval for the regression estimate.

Using the Genome-wide Complex Trait Analysis (GCTA) software (Yang et al., 2011), SNP heritability for SC-FC coupling was estimated across 3,726 vertices, with a linear mixed-effects model accounting for relatedness (Fig. 1a,1bandSupplemental Table S1). The average heritability (h^2^) estimate was 16.89% (median h^2^: 17.06%; standard error = 8.84%), comparable to the h^2^for FC and SC individually in previous studies (Elliott et al., 2019;Ge et al., 2017;Miranda-Dominguez et al., 2018;Zhao, Li, Yang, et al., 2021;Zhao, Zhang, et al., 2021). Significant heritability (mean h^2^: 33.92%; h^2^range = 28.90% to 46.36%; P < 1.34 × 10^-5^) was found in 246 vertices, and 81 exceeded 35% heritability. These were in the dorsal-attention, language, and somatomotor functional networks (mean h^2^: 18.46%, 18.12%, 17.72%, respectively,Fig. 1d). The dorsal stream visual area in the early and intermediate visual cortex had the most heritable coupling (mean h^2^: 22.08%, h^2^range = 18.29% to 24.76%,Fig. 1c), aligning with previous evidence of heritability in SC-FC coupling within the visual cortex (Gu et al., 2021).

To demonstrate that SC-FC coupling can provide additional genetic information compared to SC or FC individually, we estimated the h^2^of both SC and FC node strength (l1-norm of each row) with the same approach and calculated the Pearson’s correlation for h^2^between SC or FC node strength and coupling. The h^2^correlation between FC node degree and coupling was 0.3519 (P < 0.0001), significantly higher than SC node degree and coupling (r = 0.0237, P = 0.15). This reflected that SC-FC coupling heritability was more linked to FC, consistent with the findings inGu et al. (2021). The low to moderate correlations suggested SC-FC coupling’s distinctiveness as a heritable endophenotype. Additionally, 25.66% of coupling traits had higher heritability than FC node degree (14.35% for SC), emphasizing the unique genetic bases of SC-FC coupling. Overall, the analysis supports genetic control of SC-FC coupling across the brain, revealing specific network locations with a stronger alignment to global genetic impact.

### 3.2 Shared genetic loci between SC-FC coupling and complex traits/disorders

In a genome-wide association study utilizing HCP-YA participants, 367 distinct associations were identified between SNPs and SC-FC coupling across 3,726 vertices. These associations encompassed 334 SNPs within 234 genomic regions (cytogenetic bands) specific to 61 SC-FC coupling vertices (refer toSupplemental Table S2,Supplemental Fig. S1, andSupplemental Fig. S2), at a significance level of 1.26 × 10^−11^(0.05 / (1,064,964 × 3,726).

**Fig. 2.**
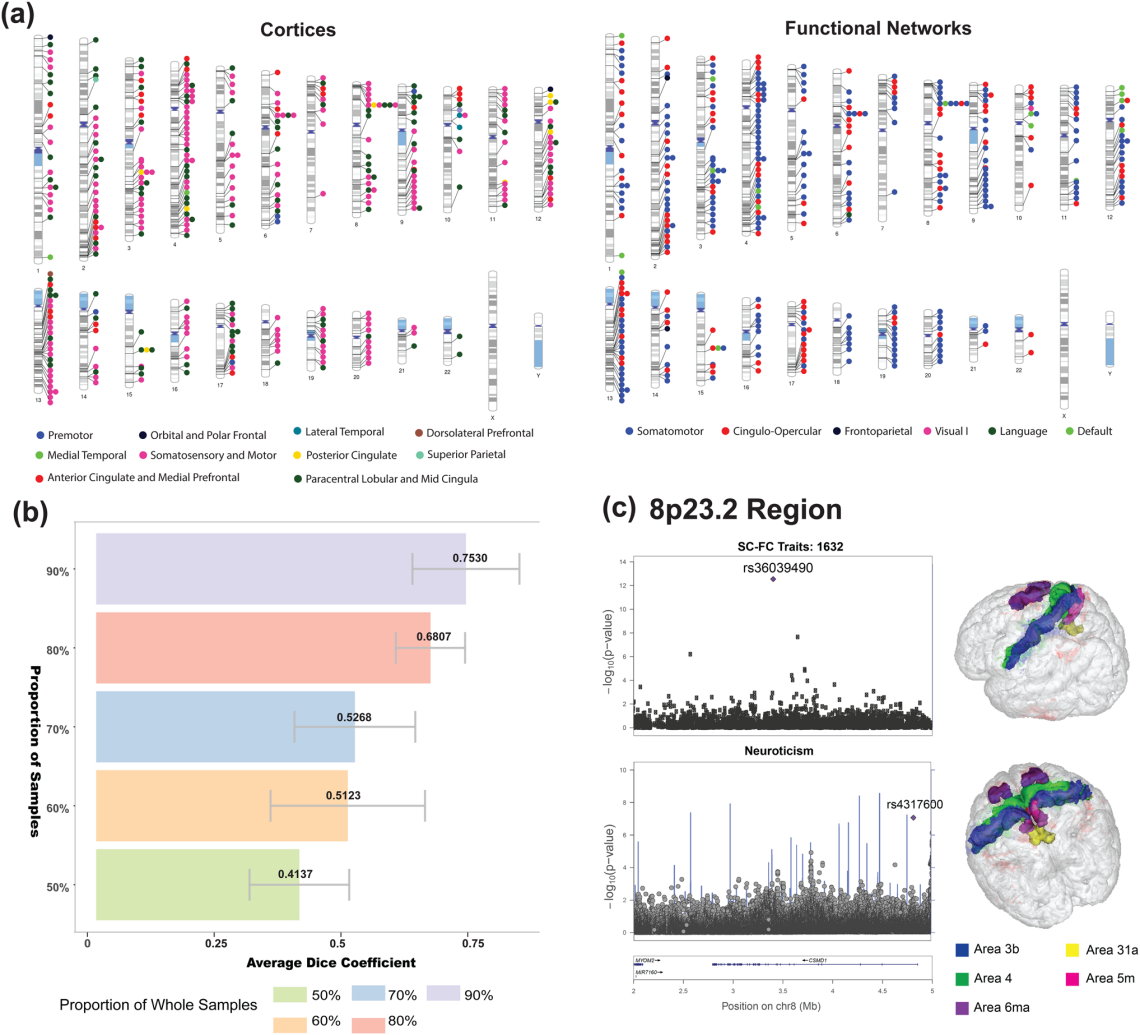
The associated genomic regions of the SC-FC coupling traits. (a) Ideogram of genomic regions influencing SC-FC coupling traits. The colors represent the 22 cortex areas (left) and 12 brain functional networks (right). Each signal point indicates that at least one of the SC-FC coupling traits within this network or cortex area is associated with the genomic region. (b) Robustness of the genetic results with respect to different sample sizes measured by dice coefficient, defined as the overlap SNP-trait associations in the subset and the whole dataset. Different colors represented different proportion of subsets, ranging from 90%–50%. The average dice coefficient was annotated on the top of each bar, and the grey interval represented mean ± SD. (c) Functional areas associated with the 8p23.2 region (HCP: P < 9.32 × 10^-12^, ABCD: P = 4.31 × 10^-7^). Most of these areas were in the primary sensory cortex (3b, blue) with few in the posterior cingulate (31a, yellow), primary motor cortex (area 4, green), paracentral lobular and mid cingulate cortex (area 5 m, pink; area 6ma, purple). The color represents regions in glasser360 atlas (red: 23c, blue: 3b, yellow: 31a, 4: green, 5 m: pink, 6ma: purple). The zoom plot of the significant locus in each plot was visualized using LocusZoom (https://statgen.sph.umich.edu/locuszoom/).

Utilizing the Glasser360 atlas, comprising 12 functional and 5 anatomical networks pertinent to SC-FC coupling, we evaluated the enrichment of SC-FC coupling across 61 vertices, mapped to 29 Glasser360 regions (seeFig. 2a). The results indicated that SC-FC coupling at these vertices was notably enriched in cingulo-opercular ($\chi^{2}$= 4.8392, uncorrected P = 0.0278) and somatomotor ($\chi^{2}$= 45.2887, uncorrected P < 0.0001) functional networks. Within anatomical networks, enrichment was observed in sensorimotor areas ($\chi^{2}$= 78.6074, uncorrected P < 0.0001), early and intermediate visual cortex ($\chi^{2}$= 8.9978, uncorrected P = 0.0027), and posterior cortex ($\chi^{2}$= 3.8557, uncorrected P = 0.0496). Locus-specific genetic effects were also found to enrich SC-FC coupling in various networks, such as in the 13q14.11 and 10p14 genomic regions ($\chi^{2}$= 6.3860, uncorrected P = 0.0115) within the cingulo-opercular network, and the 10p11.21 genomic region ($\chi^{2}$= 27.912, uncorrected P < 0.001) within the default mode network (Fig. 2a). These results emphasize the heterogeneity of genetic influences on SC-FC coupling, shedding light on its potential as a valuable measure for understanding genetic foundations.

We then explored the influence of these genetic variants on gene expressions through human brain expression quantitative trait loci (eQTL) datasets (Consortium, 2020) integration, aiming to reveal potential biological mechanisms. Several eQTLs were identified, notably in the 2p22.2 locus, influencing SC-FC traits such as the expression of the FEZ2 gene (detailed inSupplemental Figs. S3-S5).

Finally, to ascertain the robustness of our findings, we conducted internal and external validations. The internal validation, using different sample proportions ranging from 50% to 90%) from the entire dataset, affirmed consistency in SNP-vertex associations, with Dice coefficients mainly above 0.5 (Fig. 2bwith further details inSupplemental Files). The external validation was performed with the Adolescent Brain Cognitive Development (ABCD) study cohort (n = 1,546), confirming 187 cytogenetic bands associated with SC-FC coupling in 22 Glasser360 regions at a significance level of 1× 10^-5^. We reported the minimum P-value of SNPs within each cytogenetic region and the corresponding SNPs inSupplemental Table S3. The congruent magnitude of P-values between HCP and ABCD datasets strengthened our findings, offering valuable insights into the genetic basis of SC-FC coupling (Supplemental Fig. S6). For instance, SNP rs75606009 in the 8p23.2 region achieved a P-value of 1.86 × 10^-12^with Glasser360 region 4 in the HCP dataset, and another SNP, rs28570092, in the same region had a P-value of 6.73 × 10^-9^in the ABCD dataset.

In evaluating shared genetic influences between SC-FC coupling and complex traits, we conducted association lookups for significant variants within cytoband regions using the NHGRI-EBI GWAS catalog (Buniello et al., 2019), identifying multiple variants across diverse trait domains (Supplemental Table S4). A key finding included colocalizations in the 8p23.2 region where SC-FC coupling traits were primarily situated in the somatosensory and motor cortex (Glasser360 3b), with a few in the posterior cingulate (Glasser360 31a), paracentral lobular, and mid cingulate cortex (Glasser360 5 m, 6ma;Fig. 2c). Variants in this region, such as index SNP rs36039490, were reported to be associated with neuroticism (Davies et al., 2018;Nagel et al., 2018;Wendt et al., 2021) and schizophrenia (Ikeda et al., 2019;Lam et al., 2019;Trubetskoy et al., 2022). Neuroticism, as a fundamental personality trait, has been used to assess mental disorders such as anxiety disorders, major depressive disorder, psychosis, and schizophrenia (Jeronimus et al., 2016). We hypothesize that a shared genetic influence might link SC-FC decoupling with neural dysfunction in the cingulate cortex, suggesting a connection to brain atrophy in neuroticism. Additional significant relationships were identified, including effects on SC-FC coupling in specific regions linked with the index variants rs112895196, rs58942603, and rs498516 (details in Fig. S7-S9). In summary, these findings demonstrate that SC-FC coupling exhibits genetic associations with brain-related traits and disorders, underlining the importance of exploring the genetic foundations of SC-FC coupling for a nuanced understanding of the structural and functional brain mechanisms associated with complex traits.

### 3.3 Genetic correlations in cross-sectional SC-FC coupling across brain regions

**Fig. 3.**
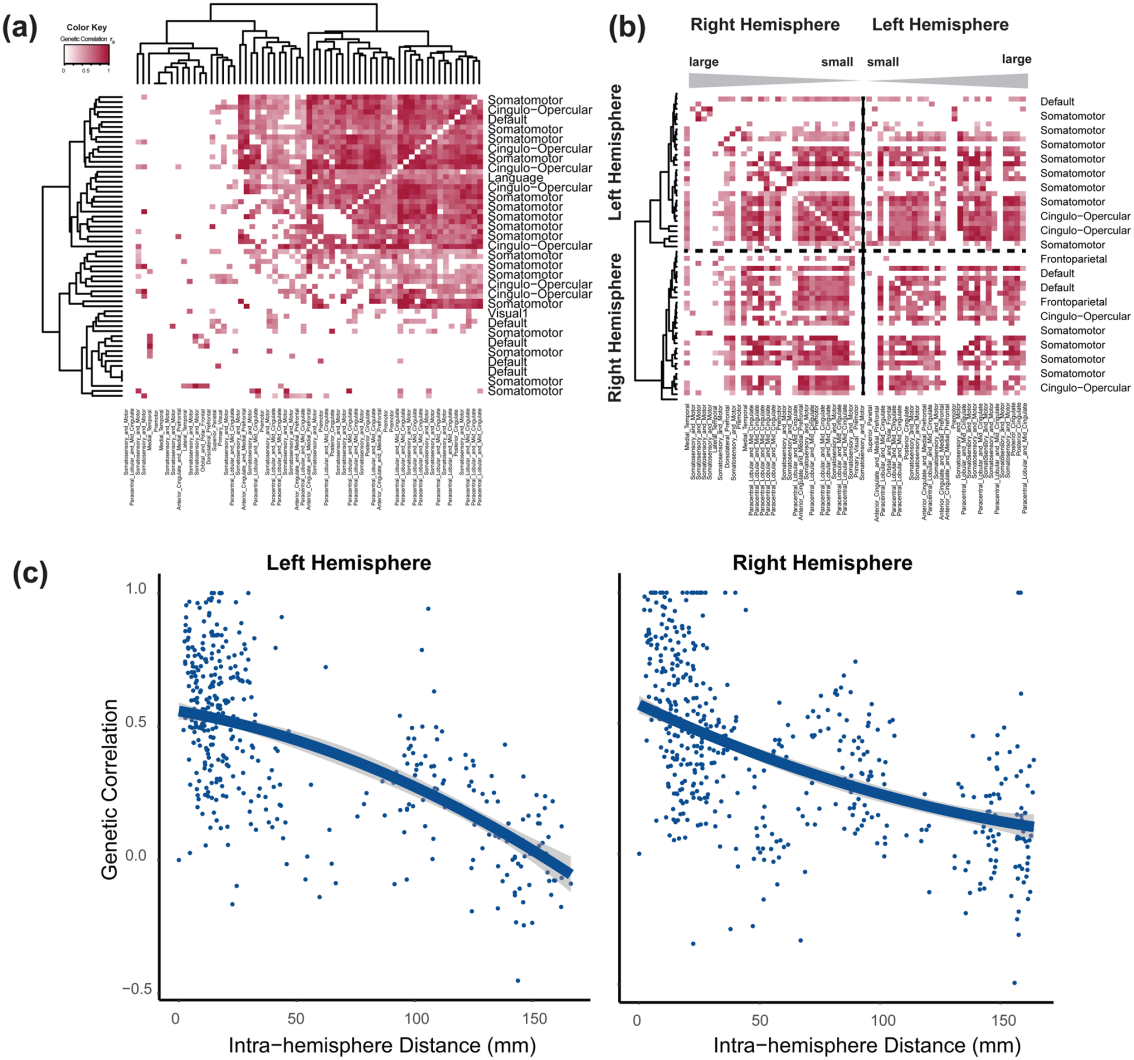
Selected pairwise genetic correlations between the SC-FC coupling traits. (a) The ordered heatmap of pairwise genetic correlations is presented by hierarchical clustering boxes, where colored boxes signify significant genetic correlations after controlling the false discovery rate at the 0.05 level. The x-axis and y-axis represent vertex-level associations with a coupling trait. This trait is then linked to corresponding functional networks and anatomical cortices based on their physical locations, respectively. The colors represent the strength of genetic correlations. (b) The ordered heatmap of pairwise genetic correlations is organized by the Euclidean distance of SC-FC coupling traits on the brain. The map is divided into left and right hemispheres. Rows and columns are ordered by Euclidean distance in each hemisphere from large to small, as indicated by the grey bar in the plot. Colored boxes highlight significant genetic correlations after controlling the false discovery rate at the 0.05 level. The x-axis and y-axis list the associated functional networks and anatomical cortex, respectively. The colors represent the strength of genetic correlations. (c) The relationship between genetic correlation and anatomical distance is depicted. The left-hand plot illustrates the relationship between anatomical distance and genetic correlation in the left hemisphere. Lines represent the fit of a quadratic model, with standard error silhouettes (ggplot2 package in R), which provides a better fit than either a linear model or an exponential decay model (Results). The right-hand plot focuses on the genetic correlation in the right hemisphere with distance. The 95% CI of fitted lines are displayed in the light blue band.

To understand shared genetic influences on SC-FC coupling across brain regions, we evaluated pairwise genetic correlations (gc) among 61 vertices (29 in the left hemisphere (LH), 31 in the right hemisphere (RH)), utilizing significant SNPs (Fig. 3a). We identified 819 significant pairs (FDR 5%, |gc| range = (0.0004, 0.9972), P range = (8.97 × 10^-208^, 0.9985)), involving regions from different functional and anatomical networks, with the somatomotor network being most represented (LH: 14; RH: 20).

The genetic correlations were inversely influenced by the anatomical distance between brain regions (Fig. 3band3c), fitting a quadratic model for intrahemispheric correlations (residual difference in the sum of squares compared with a linear model = 0.3845,$\chi_{\text{df}\;=\;1}^{2}$, P = 0.0127). Specific functional and anatomical networks displayed unique relationships between genetic correlation and distance, with the cingulo-opercular network showing stronger negative relationships, and others like the somatomotor network exhibiting weaker correlations. Some networks, such as the default network, diverged in their relationship strength between hemispheres. These results highlight the genetic correlation of SC-FC coupling in proximal brain regions, diminishing as anatomical distance increases.

### 3.4 Genetic correlations with complex traits

**Fig. 4.**
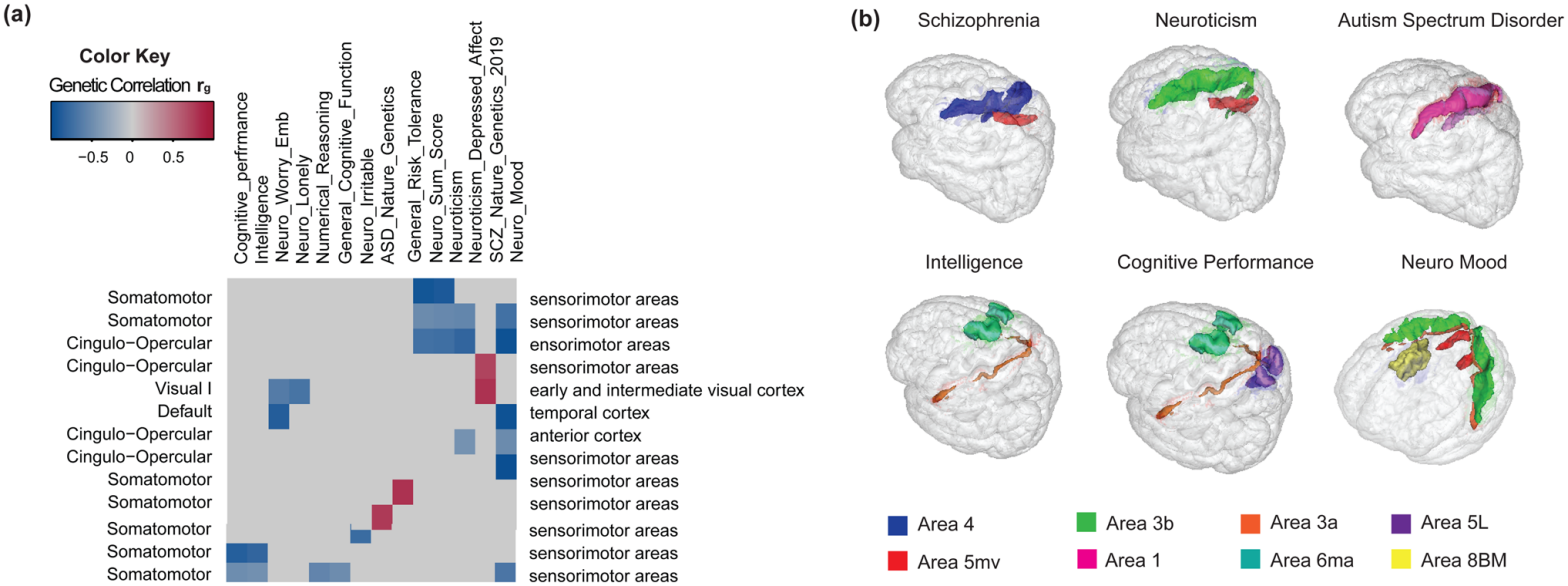
Selected pairwise genetic correlations between SC-FC coupling traits and other complex traits / brain-related disorders. (a) Significant genetic correlations (P < 0.01) between SC-FC coupling traits and other complex traits/brain-related disorders are highlighted with colored boxes. The y-axis lists the associated functional and anatomical networks of SC-FC traits, and the x-axis provides the name of the complex traits/disorders. The y-axis represents the vertex-level association with a coupling trait, which is then mapped to the corresponding region within the Glasser360 atlas, based on its physical location The colors indicate the strength of genetic correlations. (b) Locations of Glasser360 regions whose SC-FC coupling traits are genetically correlated with schizophrenia (SCZ), neuroticism, autism spectrum disorder, intelligence, cognitive performance, and neuro mood. The colors represent different Glasser360 regions.

Subsequently, we analyzed the genetic correlations between the SC-FC coupling across 61 significant vertices and 36 other complex traits, primarily focusing on brain disorders and cognitions (Supplemental Table S5). We uncovered 30 correlated pairs involving 14 complex traits, and SC-FC coupling of 14 vertices with P < 0.01 (Fig. 4a). The SC-FC coupling was found to be linked with neuroticism-related disorders, autism spectrum disorder, schizophrenia, and general risk tolerance behaviors (Fig. 4b). Notably, the strongest genetic correlation was observed with mood disorders within the context of neuroticism (gc = -0.7196, P = 7.72 × 10-4), particularly involving vertices within the somatomotor and visual I networks. This negative correlation indicates that genetic influences leading to reduced coupling values were associated with an augmented risk of mood disorders. Existing research corroborates this finding, elucidating altered neural responses in emotional processing circuits among patients with major depressive disorder and showing evidence of white matter tracts involvement, such as the left inferior frontal-occipital fasciculus (IFOF), in processing visuospatial information and facial emotions (Catani et al., 2002;Jenkins et al., 2016;Surguladze et al., 2005).

In essence, our findings underscore shared genetic underpinnings between SC-FC coupling traits and a range of complex traits and diseases, providing valuable insights that may contribute to the early identification and targeted intervention of associated mental disorders.

### 3.5 Gene-level analysis and biological annotations results

Gene-based association analysis was performed for 18,796 protein-coding genes using MAGMA (de Leeuw et al., 2015) (version 1.07). Default MAGMA settings were used with zero window size around each gene. We then carried out FUMA functional annotation and mapping analysis, in which variants were annotated with their biological functionality and then were linked to 35,808 candidate genes by a combination of positional and eQTL mappings. Brain-related tissues/cells were selected in all options, and default values were used for all other parameters in FUMA. For the detected genes in MAGMA and FUMA, we performed lookups in the NHGRI-EBI GWAS catalog (version 2022-01-12) to explore their previously reported gene-trait associations. We also performed gene property analysis for the 13 GTEx v8 (Consortium, 2020) brain tissues via MAGMA. Specifically, we examined whether the tissue-specific gene expression levels can be linked to the strength of the gene-trait association. MAGMA was also used to explore the enriched biological pathways, in which we tested 500 curated gene sets and 9,996 Gene Ontology (GO) terms from the Molecular Signatures Database (MSigDB, version 7.0) (Liberzon et al., 2011). The Gene Ontology (GO) pathway annotation database is publicly available athttps://www.gsea-msigdb.org/gsea/msigdb/.

Using GWAS summary statistics of the SC-FC coupling traits, MAGMA detected 50 significant gene-trait associations (P < 2.87 × 10^-6^, adjusted for 17, 447 genes) for 39 significant genes with 20 SC-FC coupling traits (Supplemental Table S9). Among them,*DPYSL4*was associated with cortical thickness (Shadrin et al., 2021),*TMBIM6*was related to general cognitive ability (Davies et al., 2018),*MLLT1*was reported to be associated with oppositional defiant disorder dimensions in attention-deficit hyperactivity disorder (Brevik et al., 2016), and*BAP1*was associated with autism spectrum disorder or schizophrenia (Autism Spectrum Disorders Working Group of The Psychiatric Genomics, 2017). Gene*DPYSL4*was involved in the nervous system development, neuronal death, and neuronal projection guidance pathways with functions in axon guidance, neuronal growth cone collapse, and cell migration (Parent et al., 2017;Riva et al., 2016). Gene*BAP1*encodes protein to help control cell growth and division (proliferation) and cell death by removing ubiquitin. One recent study found that rare germline missense*BAP1*variants could alter chromatin remodeling by abnormal histone ubiquitination and lead to a neurodevelopmental disorder (NDD) (Kury et al., 2022).*MLLT1*is an essential gene during development (Doty et al., 2002) and regulates histone*H3K79*demethylation (Mueller et al., 2007). A potentially deleterious homozygous variant in*MLLT1*would potentially result in developmental delay, hypotonia, infantile spasm, and cortical dysgenesis (Charng et al., 2016;Frescas et al., 2007;He et al., 2008). The identified genes provide more insights into the genetic overlaps among brain structure and brain-related disorders.

We also observed many pleiotropic genes associated with alcohol consumption, blood metabolite and protein levels, diastolic blood pressure, asthma, lymphocyte counts, obesity-related traits, serum metabolite levels, and triglyceride, all of which might be related to the major depressive disorders (Ahmedani et al., 2013;McHugh & Weiss, 2019;Rubio-Guerra et al., 2013;Watson et al., 2021;Zheng et al., 2016) and ADHD (Mogensen et al., 2011;Putz-Anderson et al., 1983;L. J. Wang et al., 2021). These results expand the scope of the shared genetic components among metabolic dysfunction, blood biomarkers, brain structure, and function coupling traits in neurodegenerative and neuropsychiatric disorders research, suggesting the potential value of integrating these traits in future studies.

Next, we performed MAGMA tissue-specific gene property analysis for 13 GTEx v8 (Consortium, 2020) brain tissues (Online Methods). We found that genes with higher expression levels in human brain tissues were primarily in the spinal cord cervical and substantia nigra (P < 0.05,Supplemental Fig. S13,Supplemental Table S10). The substantia nigra is a region in the midbrain that is considered part of the basal ganglia. Evidence has shown that altered dopaminergic neurotransmission in neurons in the substantia nigra pars compacta is one of the mechanisms of hyperactivity disorders, such as ADHD (Gallo & Posner, 2016). These results could advance the potential value of integrating coupling traits in future studies of the hyperactivity disorders including ADHD.

Finally, MAGMA gene-set analysis was performed to prioritize the enriched biological pathways (Online Methods). We found 21 significantly enriched gene sets after Bonferroni adjustment for 15,482 GO pathways (P < 3.2 × 10^−6^,Supplemental Table S11). For example, the pathway “go regulation of translation in response to stress” (GO: 0043555, P = 8.27 × 10^−7^) has been identified which was known to modulate the frequency, rate, or extent of translation due to stimulus under stress (Caspi et al., 2018).

### 3.6 Sensitivity analysis

**Fig. 5.**
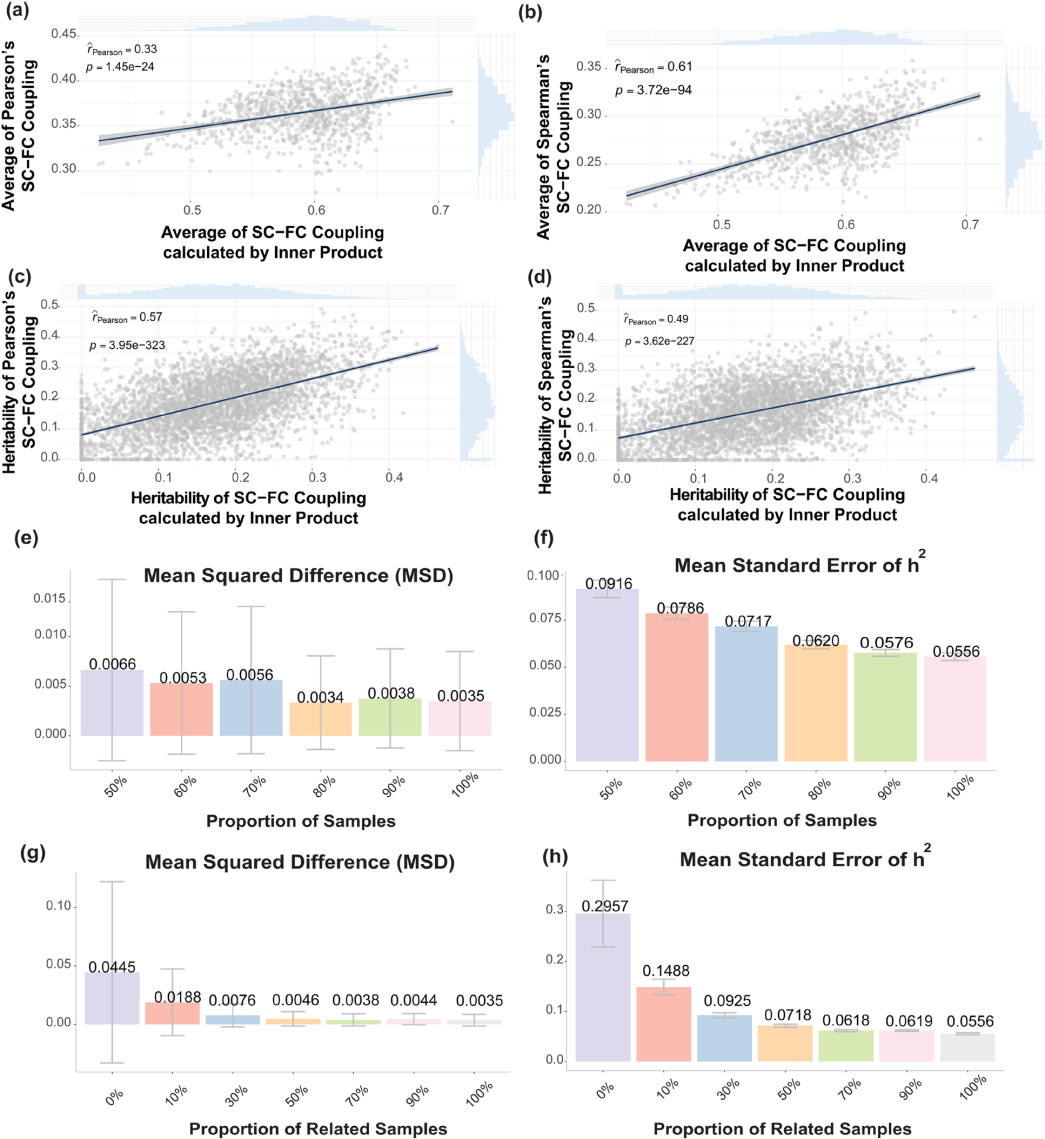
Sensitivity analysis results. (a) and (b) Relationship between the SC-FC coupling traits calculated from inner-product process (x-axis) and Pearson/Spearman process (y-axis). Higher correlation between the Spearman SC-FC coupling and inner-product SC-FC coupling (r = 0.61, P < 0.001) than that between the Pearson SC-FC and inner-product SC-FC coupling (r = 0.33, P < 0.001) demonstrated that our construction of SC-FC coupling was similar to the Spearman SC-FC coupling. (c) and (d) Relationship between the heritability estimation of SC-FC coupling traits calculated from inner-product process (x-axis) and Pearson (a) / Spearman (b) process (y-axis). The h^2^of inner-product SC-FC coupling was positively correlated with that of Pearson SC-FC coupling (r = 0.57, P < 0.001), and a similar trend was observed for Spearman SC-FC coupling (r = 0.49, P < 0.0001). (e)—(h) Simulation results on the sensitivity analysis of sample size and relatedness. Mean squared difference (MSD) =$\frac{\sum {\left({\hat{h}}^{2}-h_{true}^{2}\right)}^{2}}{\#iterations}$. Mean standard errors of h^2^=$\frac{\sum SE({\hat{h}}^{2})}{\#iterations}$. (e) and (g) Mean squared difference and mean standard errors of h^2^across different proportions of whole samples where we randomly selected a certain proportion of the samples ranging from 50% to 90% from the whole dataset. (f) and (h) Mean squared difference and mean standard errors of h^2^across different proportions of related samples where we adjusted the relatedness level by first only including unrelated samples (one from each family) and gradually adding 10 to the largest of 90% related samples.

To validate the robustness of our heritability estimation and GWAS results, we conducted sensitivity analyses considering various methods for calculating SC-FC coupling, sample sizes, and relatedness. By recalculating SC-FC coupling using both Pearson’s and Spearman’s correlation, we found consistent heritability estimations across the inner-product (current one), Pearson, and Spearman approaches (Fig. 5). The h^2^of inner-product SC-FC coupling was positively correlated with that of Pearson’s SC-FC coupling (r = 0.57, P < 0.0001), and a similar trend was observed for Spearman SC-FC coupling (r = 0.49, P < 0.0001). These results support that the way we constructed coupling was plausible and could lead to robust and reliable results.

To evaluate potential effects of limited samples and relatedness on the heritability estimation, we further performed two sets of simulations which we could evaluate over ground truth. Our aims were to demonstrate that: (1) the heritability estimation was robust and consistent under our current analyses even with a relatively limited sample size; (2) with related samples included, the standard errors of heritability estimation would be smaller, leading to a higher confidence. The first analysis was performed by randomly selecting a proportion (a grid of 50% to 90%) of samples from the whole dataset (n = 899) to simulate phenotype with the real genotype data and recalculate the heritability. To further evaluate the impact of relatedness, the second analysis was to simulate phenotype with the real genotype data and recalculate the heritability by adjusting the relatedness level from unrelated to fully related in the dataset (Materials and Methods). For both analyses, we calculated the average squared difference${\left({\hat{h}}^{2}-{\text{h}}_{\text{true}}^{2}\right)}^{2}$and average standard error of${\hat{h}}^{2}$across 4,000 iterations. FromFigure 5e–5handSupplemental Table S6, we observed that with only 50% samples, the heritability estimation was still close to the true value (mean squared difference (MSD) = 0.0066), indicating that the estimation provided relatively precise results. Consistent with our expectation, standard errors reduced with increased sample size (50% dataset: 0.0916; full dataset: 0.0556). Thus, we tried to include as many samples as possible in our analyses. With respect to the inclusion of related samples, small MSD (unrelatedness: 0.0445; full relatedness: 0.0035) across different proportions of related samples showed that relatedness would not bring a significant bias for the estimation. Similarly, with more samples, our confidence in the estimation were stronger as it reflected by smaller standard errors. Overall, the output of those experiments support the robustness of our analysis results.

Furthermore, we observed that the varied race/ethnicity of the 899 individuals did not have much influence on heritability estimates. We repeated the analyses on a subgroup of HCP including 625 white, non-Hispanic subjects and obtained consistent heritability patterns in SC-FC coupling (Pearson’s r = 0.87, P < 0.0001). For full-sample analyses, we included the first 10 principal component (PC) deriving from genotype data to mitigate the potential subpopulation confoundedness. The scatterplot of first two PCs showed that after PCs adjustment, the population stratification can be fairly addressed (Supplemental Fig. S10).

## 4 Discussion

In this paper, using dMRI, fMRI, and genotype data from HCP, we explored the genetic foundations linked with brain structure-function connectivity (SC-FC) coupling, and delineated the shared genetic architecture of this coupling with various complex disorders and quantitative traits. The adoption of the innovative SC-FC coupling at the white surface (the interface between white and gray matter) in a continuous manner provided us a potentially more versatile and robust method to analyze brain organization at an elevated resolution. This crafted coupling better captures the connections between structural and functional brain signals, reflecting their significance in neural development. We evidenced that SC-FC coupling was notably heritable, especially in areas within the early and intermediate visual cortex and across dorsal-attention, language, and somatomotor functional networks. Through genome-wide association analysis, we identified 334 SNPs (spread across 234 genomic regions) associated with SC-FC coupling in the HCP’s young adult cohort. Furthermore, our genetic correlation analyses disclosed that SC-FC coupling in proximate regions was genetically highly correlated. This insight is essential in comprehending how specific genes affect SC-FC coupling traits via brain anatomy. In assessing the genetics between SC-FC coupling and a broad spectrum of brain-related complex traits and ailments, we ascertained that SC-FC coupling in particular functional areas and networks was genetically associated with diverse diseases and characteristics, such as the connection between the primary motor and sensory cortex and mood disorders related to neurology.

While numerous efforts have been dedicated to exploring correlations between the structural and functional organizations of the human brain, most past work has concentrated on linking these correlations with behavior. One exception is a recent study byGu et al. (2021)that investigated the heritability of regional SC-FC coupling using predefined atlases. They revealed that regional SC-FC coupling was notably heritable throughout the brain, especially in subcortical, cerebellar/brainstem, and visual networks. This aligns with our heritability estimates for SC-FC coupling traits. The higher heritability of SC-FC coupling traits reported byGu et al. (2021)compared to our estimates could potentially be attributed to differences in trait construction and genetic relatedness matrix estimation. Specifically, we computed SC-FC coupling at a higher resolution for each vertex without needing an atlas, while they applied predetermined atlases, like the CC400 atlas. As heritability estimates of functional networks have been shown to vary with the parcellation used (Ribeiro et al., 2021), the choice of atlas may influence the heritability estimates for SC-FC coupling traits. In our estimation, we utilized an SNP-based genetic relatedness matrix instead of a pedigree-based matrix, a decision influenced by the availability of large-scale genotype data, and our aim to support GWAS. Pedigree-based estimations have been known to overestimate heritability (Yang et al., 2015), possibly explaining the higher heritability seen inGu et al. (2021). By applying the same approach asGu et al. (2021)to re-estimate heritability, we found substantial agreement between the methods (Pearson’s r = 0.70, P < 0.0001). Moreover, we verified the reliability and consistency of SC-FC coupling heritability through examining its uniformity across two resting scans within individuals. Re-estimating the heritability with a second resting-state scan from the same subject pool, the heritability estimates were highly consistent, with a mean difference of μ = -0.0103 (95% CI = (-0.1780, 0.1575)), as shown inSupplemental Figure S11.

Notably, compared with the studies byGu et al. (2021)which focused on heritability of coupling, the unique contributions of our work consisted of three parts: (1) establishing heritability for the innovative SC-FC coupling traits defined under an parcellation-free matter with reproducibility and high-resolution fine details; (2) making the first attempt to uncover the genetic underpinnings for brain structural and functional coupling through genome-wide association analysis; and (3) bridging the shared genetic basis of SC-FC couplings with complex brain disorders. We built our analyses on the parcellation-free SC-FC coupling traits to avoid arbitrarily choose a parcellation which may not be suitable for both structural and functional imaging and could significantly influence the results of heritability estimates (Messe, 2020). With the identification of genomic regions related to SC-FC coupling traits and the genetic correlation with brain-related complex traits and diseases such as links between the primary motor and sensory cortex to neuro mood, our studies suggest that this new coupling metric can be used as a potential neuroimaging biomarker to further understand how genetics influence brain structures, circuits and, in turn, cognition behavior and disorders.

There are still a few limitations in the present study. First, our study contends with limitations due to a modest sample size and acknowledges that while SC-FC coupling trait distributions largely approximate normality, minor deviations exist. The robustness of our linear mixed-effects models, as demonstrated by Q-Q plot analyses (Supplemental Fig. S14), offers a degree of resilience against these deviations. However, the lenient MAF cutoff and the sample size may elevate the risk of false positives, highlighting the need for validation in larger cohorts with robust methods to address potential non-normality. To ensure the integrity of our findings, we conducted stability analyses and external validations with the ABCD cohort, yet we opted against stringent multiple comparison adjustments at this stage to avoid overlooking true associations. Future studies with expanded cohorts and rigorous statistical adjustments are essential for confirming and refining the genetic and brain coupling correlations that we report. Second, our study used independent single-trait GWAS with Bonferroni correction. While this conservative approach helps control Type I errors, it may lack power to detect associations that multi-trait GWAS methods could capture considering potential correlation among the 3,000+ vertices analyzed. We chose the traditional single-trait approach for its clarity and robustness in this exploratory analysis. Future research could benefit from applying advanced multi-trait methods that considers spatial dependence to potentially uncover associations that our current approach may have missed. Third, we chose the Yeo7 network in our coupling formula to capture localized interactions and maintain spatial smoothness, supported by evidence of stronger genetic contributions within networks compared to between them (Arnatkeviciute et al., 2021). This method balances the trade-off between capturing genetic signals and minimizing noise. However, given the continuous nature of our SC, FC, and SC-FC coupling metrics across the cortex, future studies should explore their utility in both global and local contexts, as well as in non-parcellated and parcellated frameworks. Fourth, the functional connectivity was derived from resting-state fMRI data. Given some previous studies found that task-based functional connectivity in the secondary visual and somatomotor networks was genetically correlated with cognitive function (Zhao, Li, Smith, et al., 2021), it may be worth investigating whether task-based SC-FC coupling can further provide new insights. Besides, from previous work, SC-FC coupling can vary differently with age across the lifespan (Zamani Esfahlani et al., 2022), so the interpretations of our current findings should be restricted to young adult populations (Baum et al., 2020). Future work will generalize and validate current findings to the population at different ages and observe the dynamic genetic changes of SC-FC couplings through the life span.

## Supplementary Material

Supplementary Material

## Data Availability

The MRI imaging and most behavioral data are publicly available athttps://db.humanconnectome.org/with the acceptance of HCP Open Access Data Use Terms. Some data elements, including family structure, exact age, handedness, ethnicity, and race, are available only to qualified investigators who agree to HCP’s Restricted Data Use Terms. The genotype data are available through the dbGAP repository (https://www.ncbi.nlm.nih.gov/projects/gap/cgi-bin/study.cgi?study_id=phs001364.v1.p1). It requires application for the data access on dbGaP. The specific data used in this work are WU-Minn HCP Data-1200 Subjects. The individual-level imaging and genetics data of ABCD are from a publicly accessible data resource: ABCD (https://abcdstudy.org/). The gene expression data used for eQTL and gene property analysis across different brain tissues are publicly available athttps://www.gtexportal.org/home/. The Gene Ontology (GO) pathway annotation database is publicly available athttps://www.gsea-msigdb.org/gsea/msigdb/. Summary statistics from the GWAS are available on request from the authors.
