# Supplementary material for: Heritability and genetic contribution analysis of structural-functional coupling in human brain
Source: Imaging Neurosci (Camb). 2024 Oct 30;2:imag-2-00346. doi: 10.1162/imag_a_00346 (PMC12290780; doi:10.1162/imag_a_00346)
Supplement: Supplementary Material [file imag_a_00346-supp.zip › Supplemental_Materials.pdf]

# Supplemental Materials for

## Heritability and Genetic Contribution Analysis of Structural-Functional Coupling in Human Brain.

Wei Dai[1], Zhengwu Zhang[2], Peihan Song[1], Heping Zhang[1*], Yize Zhao[1*]

[1]Department of Biostatistics, Yale University School of Public Health, New Haven, CT 06511.
[2]Department of Statistics, University of North Carolina at Chapel Hill, Chapel Hill, NC 27599.

*Heping Zhang, *Yize Zhao
Email: heping.zhang@yale.edu, yize.zhao@yale.edu

**The supplementary materials include:**

**Supplemental Notes:**
S1. Internal validation of GWA analysis results.
S2. The eQTL analysis results.
S3. Additional results of the shared genetic loci with brain-related complex traits and disorders.
S4. Gene-level analysis and biological annotations results.
S5. Code Availability.

**Supplemental Fig 1-14**

**Supplemental Tables:**

Table S1 (separate file). SNP Heritability Estimation of 3726 SC-FC coupling, FC node degree and FC node degree traits (n = 899).

Table S2 (separate file). List of significant genetic risk loci discovered for SC-FC coupling traits at $1.26 \times 10^{-11}$ significance level (n=899 subjects).

Table S3 (separate file). Validated cytoband regions in ABCD (n=1,546) with SC-FC coupling.

Table S4 (separate file). Significant ($p < 1.26 \times 10^{-11}$) variants and their correlated variants for SC-FC coupling traits that have previously been identified in GWAS of any traits listed in the GWAS catalog (version 2022-01-02)

Table S5 (separate file). Sources of the publicly available GWAS summary statistics used in this study.

Table S6 (separate file). Simulation results of the sensitivity analysis to sample size and relatedness. (a) Mean squared difference and mean standard errors of $h^2$ across different proportions of whole samples where we randomly selected a certain proportion of the samples ranging from 50% to 90% from the whole dataset. (b) Mean squared difference and mean standard errors of $h^2$ across different proportions of related samples where we adjusted the relatedness level by first only including unrelated samples (one from each family) and gradually adding 10 - 90% related samples.

Table S7 (separate file). Demographic data for subjects in the analysis. (a) Demographic data for different family structures for n = 899 samples. Age and handedness are displayed in years: mean

± SD. MZ monozygotic twins, DZ dizygotic twins. (b) Demographic data for different family structures for n = 625 white and non-Hispanic samples. Age and handedness are displayed in years: mean ± SD. MZ monozygotic twins, DZ dizygotic twins. (c) Demographic data for n = 1546 samples in ABCD study. Age is displayed in years: mean ± SD.

Table S8 (separate file). ID of 3,726 SC-FC coupling and their map onto Glasser360 atlas, 12 functional networks, 22 cortices ad 5 anatomical networks

Table S9 (separate file). Significant ($p < 2.87 \times 10^{-6}$) genes detected by MAMGA for SC-FC coupling traits that have previously been identified in GWAS of any traits listed in the GWAS catalog (version 2022-01-02).

Table S10 (separate file). MAGMA gene property analysis for HCP GWAS results (n=899 subjects) and 13 brain tissues. The 13 brain tissues were from GTEx v8 RNA-seq database. Significant tissue groupings ($p < 0.05$) are highlighted in bold

Table S11 (separate file). Significant ($P < 3.2 \times 10^{-6}$) gene sets from MAGMA gene-set analysis to prioritize the enriched biological pathways for HCP GWAS results (n=899 subjects) after Bonferroni correction.

**SI References**

## Supplemental Notes

### S1. Internal validation of GWA analysis results

To demonstrate the robustness of the genetic results in relation to varying sample sizes, we conducted the GWA analysis with different proportions of samples. Specifically, we randomly selected a proportion (ranging from 50% to 90%) of samples from the entire dataset ten times. At each instance, we repeated the genome-wide association to identify significant SNP-trait associations and calculated the Dice coefficient to assess the agreement of significant associations across the various subsets. As depicted in Fig. 2b, substantial overlaps between the identified SNP-trait associations in the subset and the entire dataset were observed as sample sizes approached our current one. Dice coefficients were greater than 0.5, with the exception of using only 50% of samples, indicating that the identified associations were robust and likely reproducible even with limited sample sizes.

### S2. The eQTL analysis results

We investigated whether the identified genetic variants influenced gene expressions by integrating previously published human brain expression quantitative trait loci (eQTL) datasets (1). Given that most genetic loci identified through GWAS are located in non-coding regions of the genome, eQTL integration can offer potential biological mechanism interpretations for risk loci. It has been hypothesized that, in certain tissues, a SNP may affect the expression of a nearby gene, and both the gene and tissue could contribute to the disease mechanism. Our objective in this analysis was to utilize eQTL integration to uncover potential biological mechanisms of the identified SNPs. Based on our analysis, several eQTLs were identified. For instance, for all identified SNPs in the 2p22.2 locus, the corresponding SC-FC traits were in the SCEF (Glasser360 atlas) of the cingulo-opercular network, and they were in the same genomic region as eQTLs of the FEZ2 gene, including rs36048308, rs17019685, and rs75784649. Fig. 2c depicted the gene expression levels of FEZ2 and demonstrated that these eQTLs influenced the expression of FEZ2 in the brain tissue of the brain caudate basal ganglia and putamen basal ganglia. The locations of the index variant rs77476564 (and its proxy variants, linkage disequilibrium [LD] $r^2 > 0.6$) and FEZ2 were shown in Fig. S3. The low eQTL p-values in the brain tissue of the brain caudate basal ganglia and putamen basal ganglia (Fig. 2d) suggested that the identified SNPs in the 2p22.2 locus were likely to influence SC-FC coupling by affecting the gene expression of FEZ2. We also observed several other loci that were eQTLs or within the same genomic region as eQTLs with increased expression in different brain tissues (Fig. S4 and Fig. S5).

### S3. Additional results of the shared genetic loci with brain-related complex traits and disorders

The index variant rs112895196 (ENOX1) in the 13q14.11 region (Fig. S7) exhibited genetic effects on SC-FC coupling traits located in the cingulo-opercular network. Variants in this region have previously been reported to be associated with attention-deficit disorder (2), schizophrenia (2-4), autism spectrum disorder and major depressive disorder (2, 5). In a prior study, patients with major depressive disorder displayed abnormalities in the functional connectivity of the cingulo-opercular network, specifically between the dorsal anterior cingulate cortex and bilateral middle frontal gyrus (MFG), left angular gyrus (LAG), and precentral gyrus (6). Major depressive disorders not only led to functional abnormalities but also altered brain structures for both gray matter and white matter (7). Our findings highlight the shared genetic influences between SC-FC coupling and major depressive disorder.

We also identified several other relationships, including the index variant rs58942603 in the 16q21 region associated with attention deficit hyperactivity disorder (ADHD) (8) with effects on primary sensory and motor cortex, 3b and 3a (Fig. S8). It has also been reported to be associated with autism spectrum disorder (9, 10), substance use (11), brain measurement (12), cognitive decline (13) and neuroticism (14). Additionally, the index variant rs498516 in the 10p14 region has

frequently been reported to be associated with smoking behavior (15), cortical surface area (12), and left–right brain asymmetry trait (16) with effects on the anterior cingulate and medial prefrontal cortex within the cingulo-opercular network (Fig. S9).

**S4. Gene-level analysis and biological annotations results**

Gene-based association analysis was performed for 18,796 protein-coding genes using MAGMA (17) (version 1.07). Default MAGMA settings were used with zero window size around each gene. We then carried out FUMA functional annotation and mapping analysis, in which variants were annotated with their biological functionality and then were linked to 35,808 candidate genes by a combination of positional and eQTL mappings. Brain-related tissues/cells were selected in all options and default values were used for all other parameters in FUMA. For the detected genes in MAGMA and FUMA, we performed lookups in the NHGRI-EBI GWAS catalog (version 2022-01-12) to explore their previously reported gene-trait associations. We also performed gene property analysis for the 13 GTEx v8 (1) brain tissues via MAGMA. Specifically, we examined whether the tissue-specific gene expression levels can be linked to the strength of the gene-trait association. MAGMA was also used to explore the enriched biological pathways, in which we tested 500 curated gene sets and 9,996 Gene Ontology (GO) terms from the Molecular Signatures Database (MSigDB, version 7.0) (18).

Using GWAS summary statistics of the SC-FC coupling traits, MAGMA detected 50 significant gene-trait associations ($P < 2.87 \times 10^{-6}$, adjusted for 17, 447 genes) for 39 significant genes with 20 SC-FC coupling traits (Dataset S9). Among them, *DPYSL4* was associated with cortical thickness (19), *TMBIM6* was related to general cognitive ability (20), *MLLT1* was reported to be associated with oppositional defiant disorder dimensions in attention-deficit hyperactivity disorder (21) and *BAP1* was associated with autism spectrum disorder or schizophrenia (22). Gene *DPYSL4* was involved in the nervous system development, neuronal death and neuronal projection guidance pathways with functions in axon guidance, neuronal growth cone collapse and cell migration (23, 24). Gene *BAP1* encodes protein to help control cell growth and division (proliferation) and cell death by removing ubiquitin. One recent study found that rare germline missense *BAP1* variants could alter chromatin remodeling by abnormal histone ubiquitination and lead to a neurodevelopmental disorder (NDD) (25). *MLLT1* is an essential gene during development (26) and regulates histone *H3K79* demethylation (27). A potentially deleterious homozygous variant in *MLLT1* would potentially result in developmental delay, hypotonia, infantile spasm, and cortical dysgenesis (28-30). The identified genes provide more insights into the genetic overlaps among brain structure and brain-related disorders.

We also observed many pleiotropic genes associated with alcohol consumption, blood metabolite and protein levels, diastolic blood pressure, asthma, lymphocyte counts, obesity related traits, serum metabolite levels and triglyceride, all of which might be related to the major depressive disorders (31-35) and ADHD (36-38). These results expand the scope of the shared genetic components among metabolic dysfunction, blood biomarkers, brain structure and function coupling traits in neurodegenerative and neuropsychiatric disorders research, suggesting the potential value of integrating these traits in future studies.

Next, we performed MAGMA tissue-specific gene property analysis for 13 GTEx v8 (1) brain tissues (Online Methods). We found that genes with higher expression levels in human brain tissues were primarily in the spinal cord cervical and substantia nigra ($P < 0.05$, Fig. S13, Dataset S10). The substantia nigra is a region in the midbrain that is considered part of the basal ganglia. Evidence has shown that altered dopaminergic neurotransmission in neurons in the substantia nigra pars compacta is one of the mechanisms of hyperactivity disorders, such as ADHD (39). These results could advance the potential value of integrating coupling traits in future studies of the hyperactivity disorders including ADHD.

Finally, MAGMA gene-set analysis was performed to prioritize the enriched biological pathways (Online Methods). We found 21 significantly enriched gene sets after Bonferroni adjustment for

15,482 GO pathways ($P < 3.2 \times 10^{-6}$, Dataset S11). For example, the pathway “go regulation of translation in response to stress” (GO: 0043555, $p = 8.27 \times 10^{-7}$) has been identified which was known to modulate the frequency, rate or extent of translation due to stimulus under stress (40).

## S5. Code Availability

The code to reproduce the main results of this paper is publicly available at https://github.com/daiw3/Continous_SFC. The Surface-Based-Connectome-Integration (SBCI) pipeline used for constructing SC-FC coupling is publicly available at https://github.com/murrayk085/SBCI with example script to generate a coupling trait. The code for heritability, genome-wide association study and genetic correlations are through public available software Genome-wide Complex Trait Analysis (GCTA) at https://yanglab.westlake.edu.cn/software/gcta/. The code of the LME model for heritability estimation using repeated MRI scans is publicly available at https://github.com/zijin-gu/scfc-coupling/blob/main/herit_demo.m. The genetic correlation between SC-FC coupling and complex traits are estimated a publicly available software LDSC at https://github.com/bulik/ldsc. Functional mapping and annotation of genome-wide association studies, including gene-based association analysis uses a publicly available software FUMA at https://fuma.ctglab.nl/. The cytogenetic region and gene annotation of each SNP is found through a publicly available software ANNOVAR at https://annovar.openbioinformatics.org/.

**Supplemental Fig S1.** (a) Number of significant SNPs across 12 functional networks and (b) 5 anatomical networks. (a) Distributions of -log10(p) of significant SNPs for SC-FC coupling within functionally defined networks: AUD – auditory; CON – cingulo-opecular; DMN – default mode; DAN – dorsal attention; FPN – frontoparietal; LAN – language; ORA – orbito-affective; PMM – posterior multimodal; SMN – somatomotor; VMM – ventral multimodal; VIS1 – primary visual; VIS2 – secondary visual. (b) Distributions of -log10(p) of significant SNPs for SC-FC coupling within anatomically defined networks: ATC – anterior cortex; AUD – auditory regions; EIV – early and intermediate visual cortex; PTC – posterior cortex; SMA – sensorimotor areas; TPC – temporal cortex. The red dashed line represents the significance level used in GWAS, i.e. $1.26 \times 10^{-11}$ adjusted by $0.05 / 1{,}064{,}964 \times 3{,}726$.

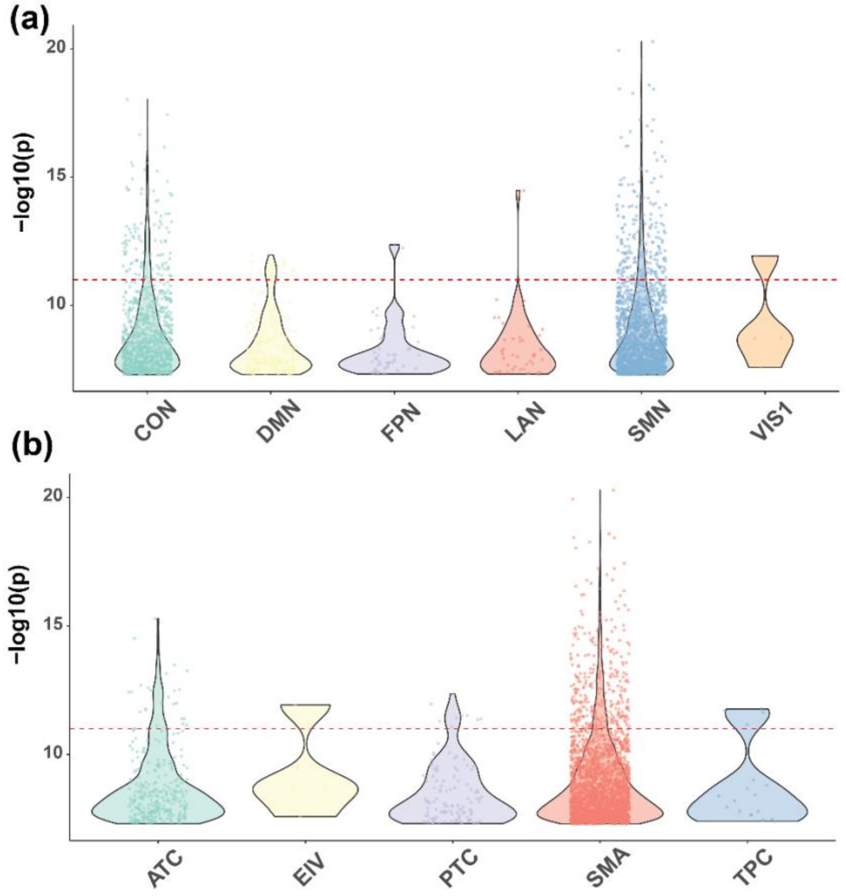

**Supplemental Fig S2.** The genome-wide association performed between 3,726 SC-FC coupling traits and 1,064,964 SNPs on a cohort of n = 899 subjects. Manhattan plots of the 8 SC-FC coupling traits that had over 10 significant SNPs. The points represent a -log10(p) of the association test between genetic variants and SC-FC coupling where higher points denote SNPs or common variants on the genome whose genetic variation is associated with SC-FC coupling in the brain.

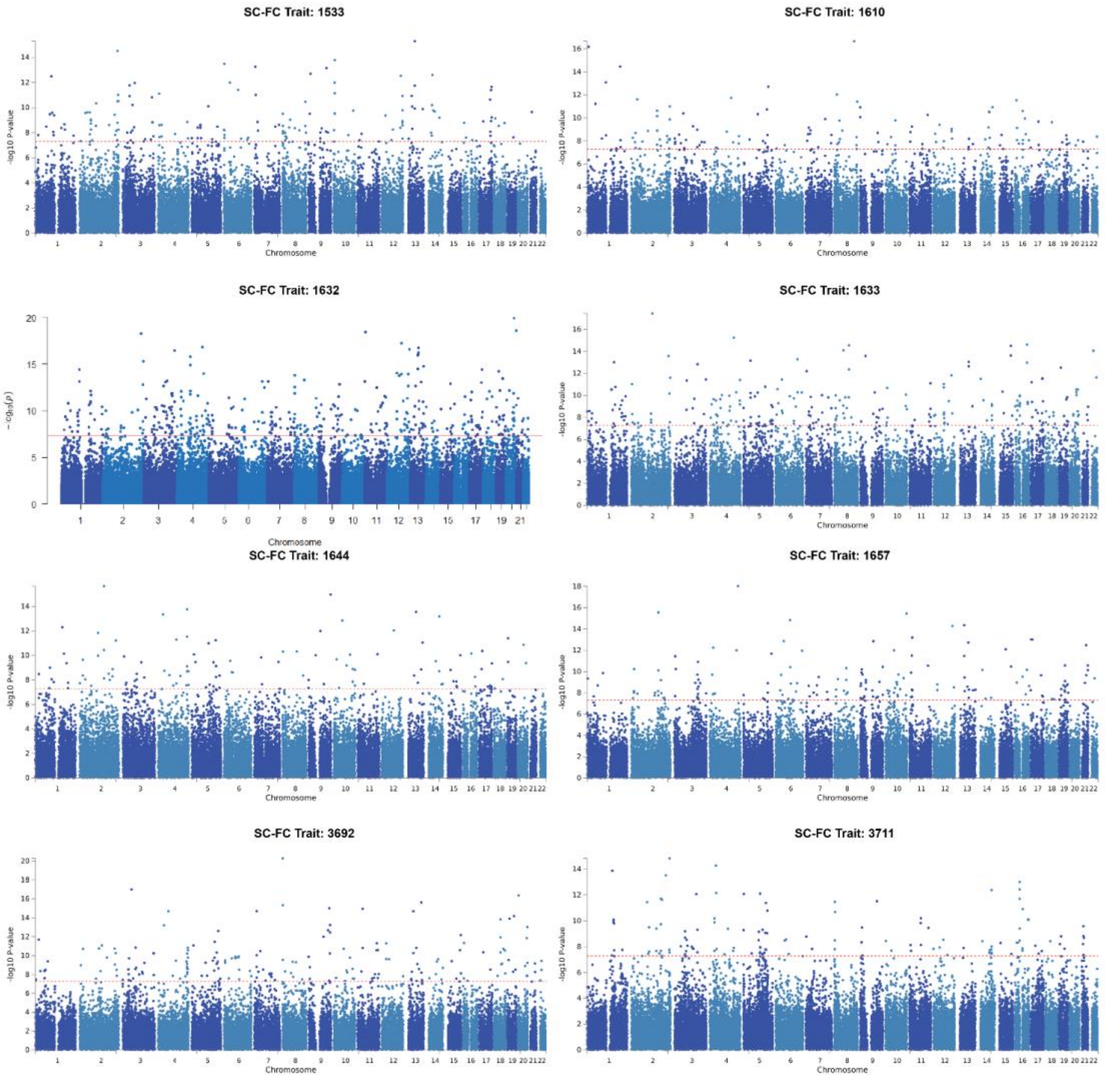

**Supplemental Fig S3.** The expression levels of FEZ2 across three eQTLs, rs36048308, rs17019685 and rs75784649, which were on the same 2p22.2 locus as index variant rs77476564 identified to be associated with one SC-FC coupling in GWAS. It showed that these eQTLs influenced the expression of FEZ2 in the brain tissue of brain caudate basal ganglia and putamen basal ganglia. (d): One SC-FC trait indexed as 1610 (in the SCEF Glasser360 atlas of the cingulo-opercular network) was associated with the 2p22.2 locus (index variant rs77476564). The index variant and its three proxy variants (LD $r^2 \geq 0.6$) are known brain eQTLs for the gene FEZ2 (colored in red). The small eQTL p-values in the brain tissue of brain caudate basal ganglia (blue dot) and putamen basal ganglia (green dot) indicated that the identified SNPs in the 2p22.2 locus were likely to influence the SC-FC coupling by affecting the gene expression of FEZ2.

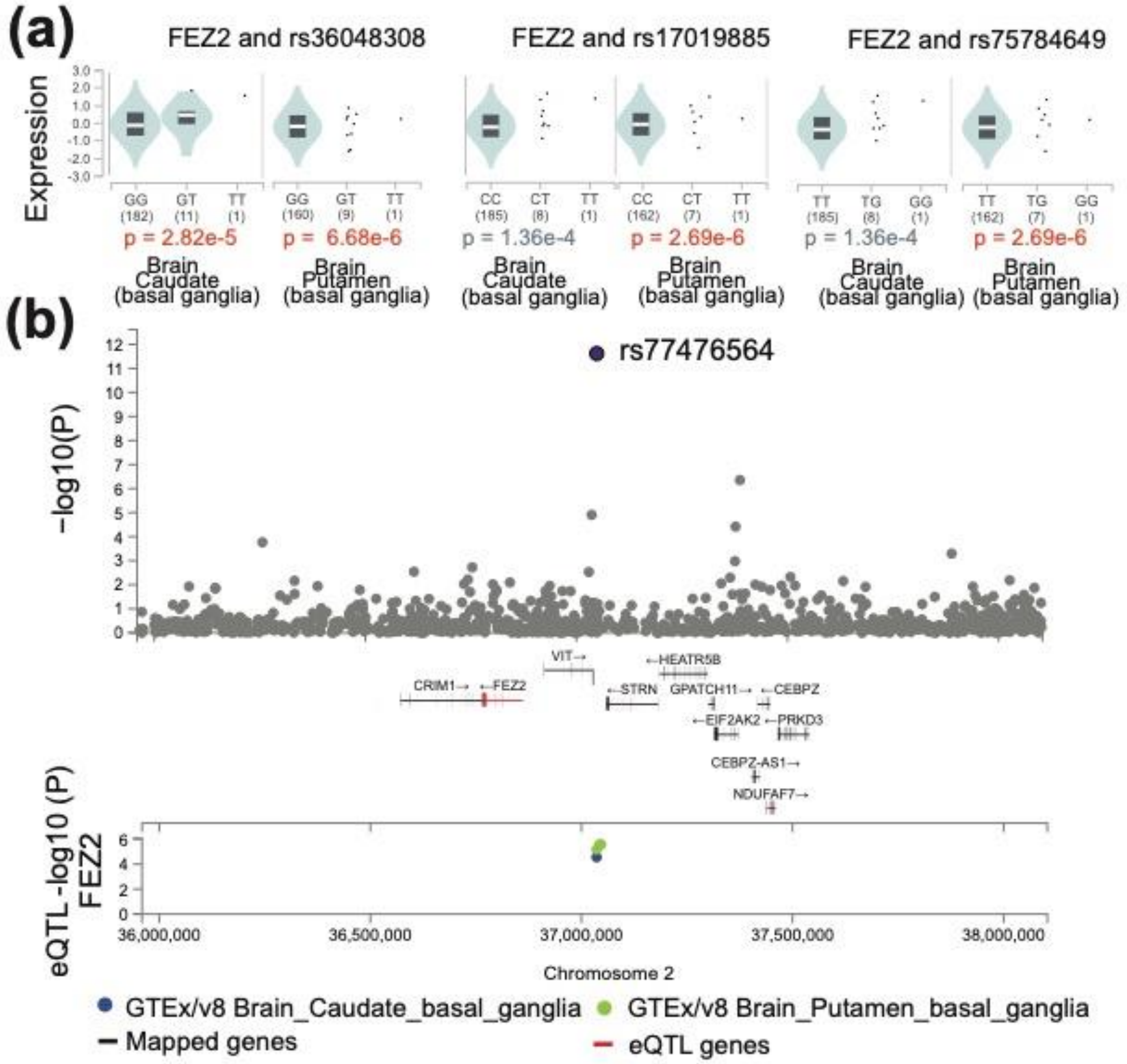

**Supplemental Fig S4.** The SC-FC trait with index of 3000 within the somatomotor network is associated with the 3q21.2 locus (index variant rs7340742). The index variant and its proxy variants (LD $r \geq 0.6$) are known brain eQTLs for the genes IFT122, and EFCAB12 (colored in red). (a): The expression levels of both genes across the eQTL rs9867650, which were on the same 3q21.2 locus as index variant rs7340742 associated with one SC-FC coupling in GWAS showed difference in the multiple brain tissues, including brain cerebellum, amygdala, etc. (b) One SC-FC trait indexed as 3000 (in the somatomotor network) was associated with the 3q21.2 locus (index variant rs7340742). The index variant and its one proxy variants (LD $r^2 \geq 0.6$) are known brain eQTLs for the IFT122, and EFCAB12 (colored in red). The small eQTL p-values in the brain tissue (dots) indicated that the identified SNPs in the 3q21.2 locus were likely to influence the SC-FC coupling by affecting the gene expression of IFT122, and EFCAB12.

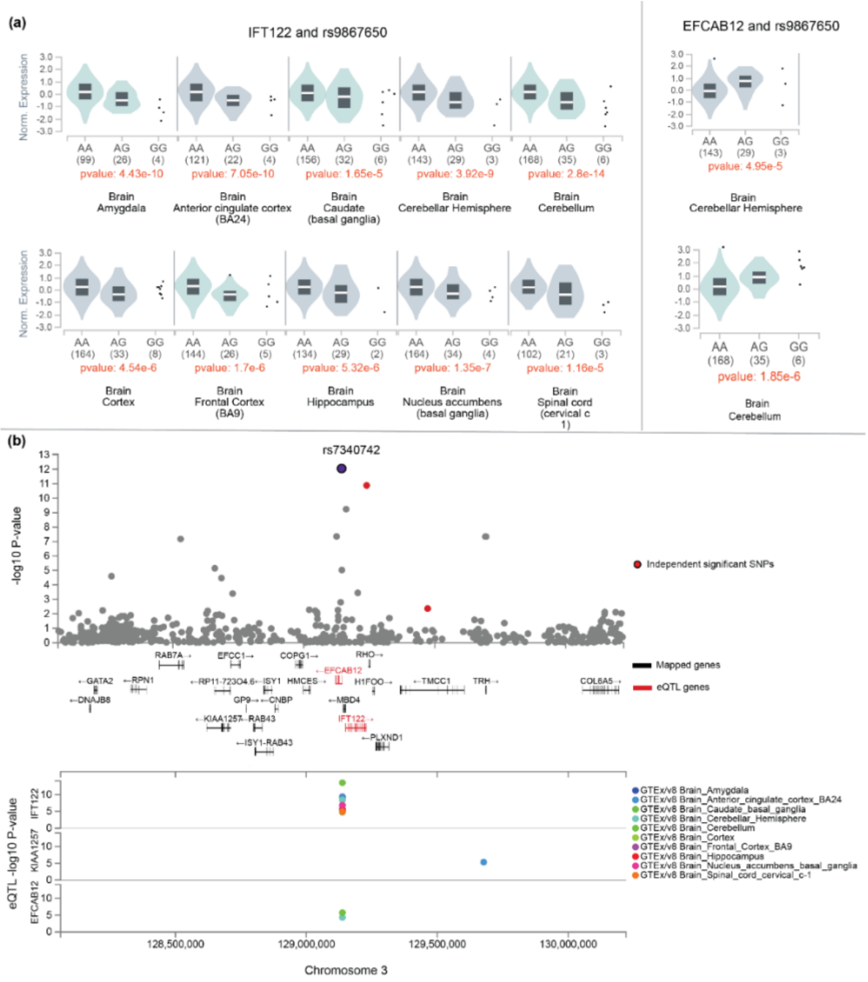

**Supplemental Fig S5.** The SC-FC trait with index of 3692 within the cingulo-opercular network network is associated with the 16p13.3 locus (index variant rs60009976). The index variant and its proxy variants (LD $r \geq 0.6$) are known brain eQTLs for the genes CIITA (colored in red). (a): The expression levels of the gene CIITA across the eQTLs rs75655915 and rs79766901, which were on the same 16p13.3 locus as index variant rs60009976 associated with one SC-FC coupling in GWAS showed difference in the multiple brain tissues, including brain nucleus accumbens (basal ganglia), amygdala, etc. (b) One SC-FC trait indexed as 3692 (in the cingulo-opercular network network) was associated with the 16p13.3 locus (index variant rs60009976). The index variant and its two proxy variants (LD $r^2 \geq 0.6$) are known brain eQTLs for the IFT122, and EFCAB12 (colored in red). The small eQTL p-values in the brain tissue (dots) indicated that the identified SNPs in the 16p13.3 locus were likely to influence the SC-FC coupling by affecting the gene expression of CIITA.

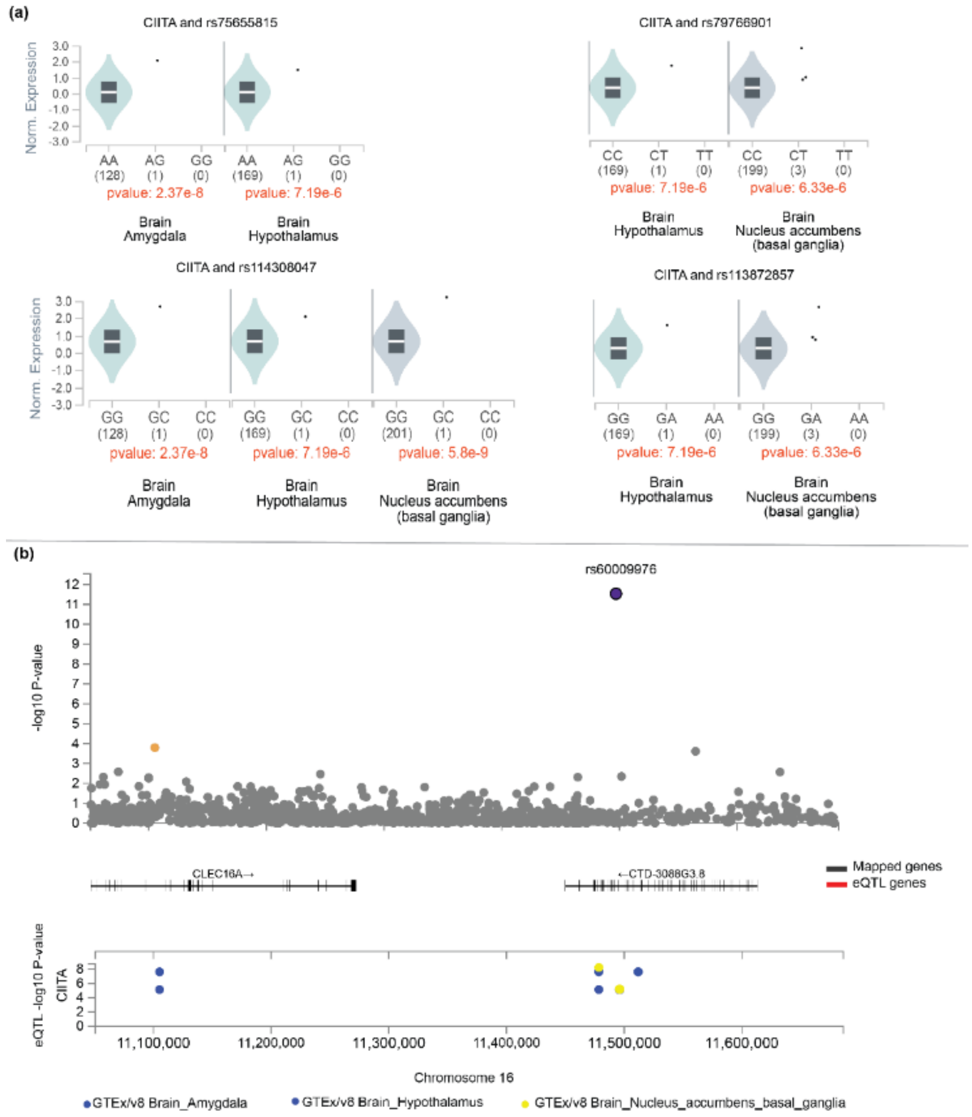

**Supplemental Fig S6.** The external validation to verify associations between SNPs in 234 cytogenetic bands and SC-FC coupling traits in 29 Glasser360 regions in the Adolescent Brain Cognitive Development (ABCD) study cohort (n = 1546). Dot plots of verified 55 cytogenetic bands associated with SC-FC coupling in the same Glasser360 regions in HCP-YA cohort. The points represent a -log10(p) of the association test between a cytogenetic band and SC-FC coupling where higher points denote SNPs or common variants on the genome whose genetic variation is associated with SC-FC coupling in the brain. Dot colors represent results from two different datasets (red for ABCD and blue for HCP).

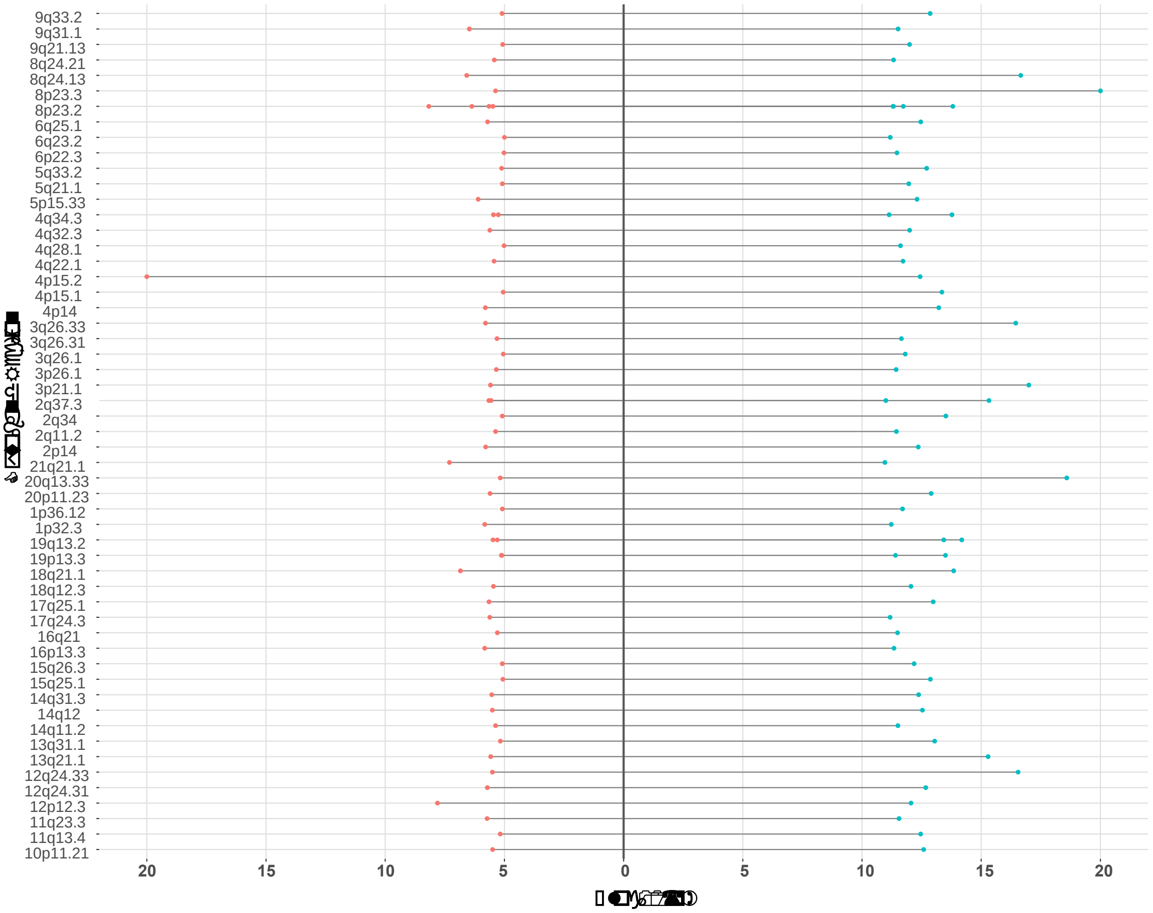

**Supplemental Fig S7.** Functional areas in the cingulo-opercular and somatomotor networks were associated with the 13q14.11 region (HCP: $P < 3.43 \times 10^{-12}$, ABCD: $P < 9.63 \times 10^{-15}$). These associations were enriched in the paracentral lobular and mid cingulate areas (23c) in the cingulo-opercular network (colored in red). The zoom plot of the significant locus in each plot was visualized using LocusZoom (https://statgen.sph.umich.edu/locuszoom/).

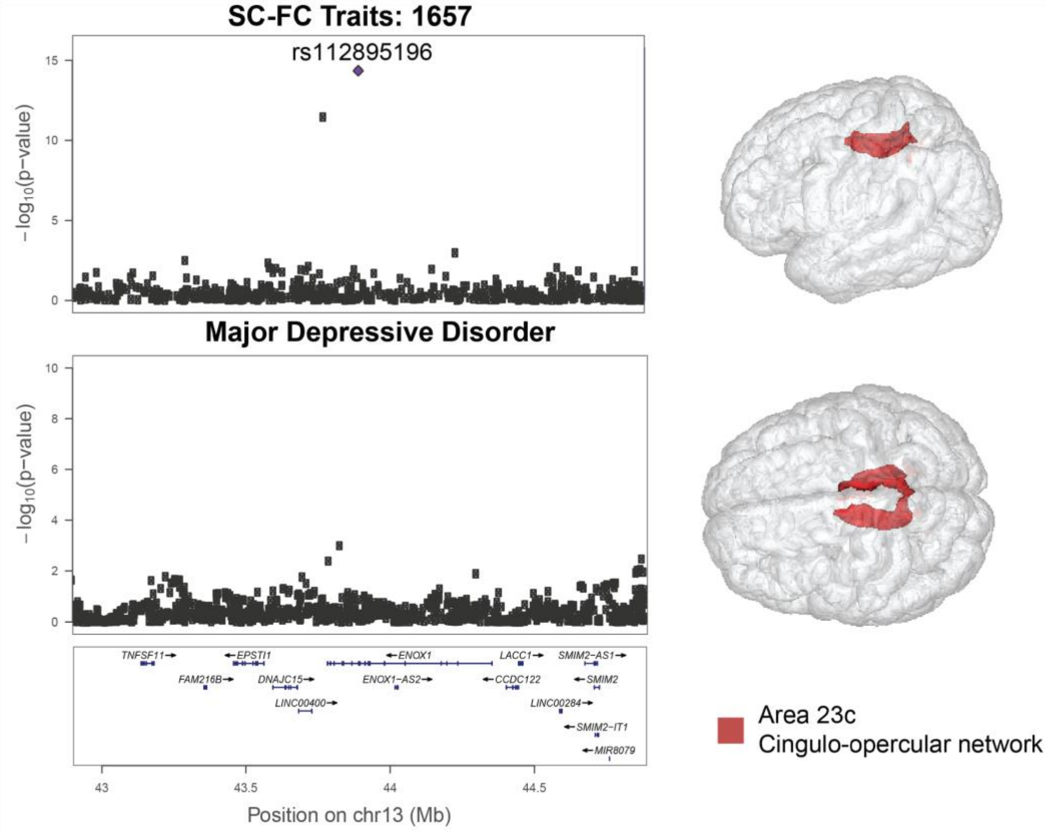

**Supplemental Fig S8.** Local colocalization between the selected SC-FC coupling trait and other brain-related complex traits/disorders in the 16q21 genomic region. Location and Glasser360 region of the displayed SC-FC coupling trait are illustrated at the bottom. Functional areas associated with the 16q21 are main in the area 3a (red) and area 3b (blue) of Glasser360 atlas. The index variant rs58942603 in the 16q21 region is associated with attention deficit hyperactivity disorder (ADHD).

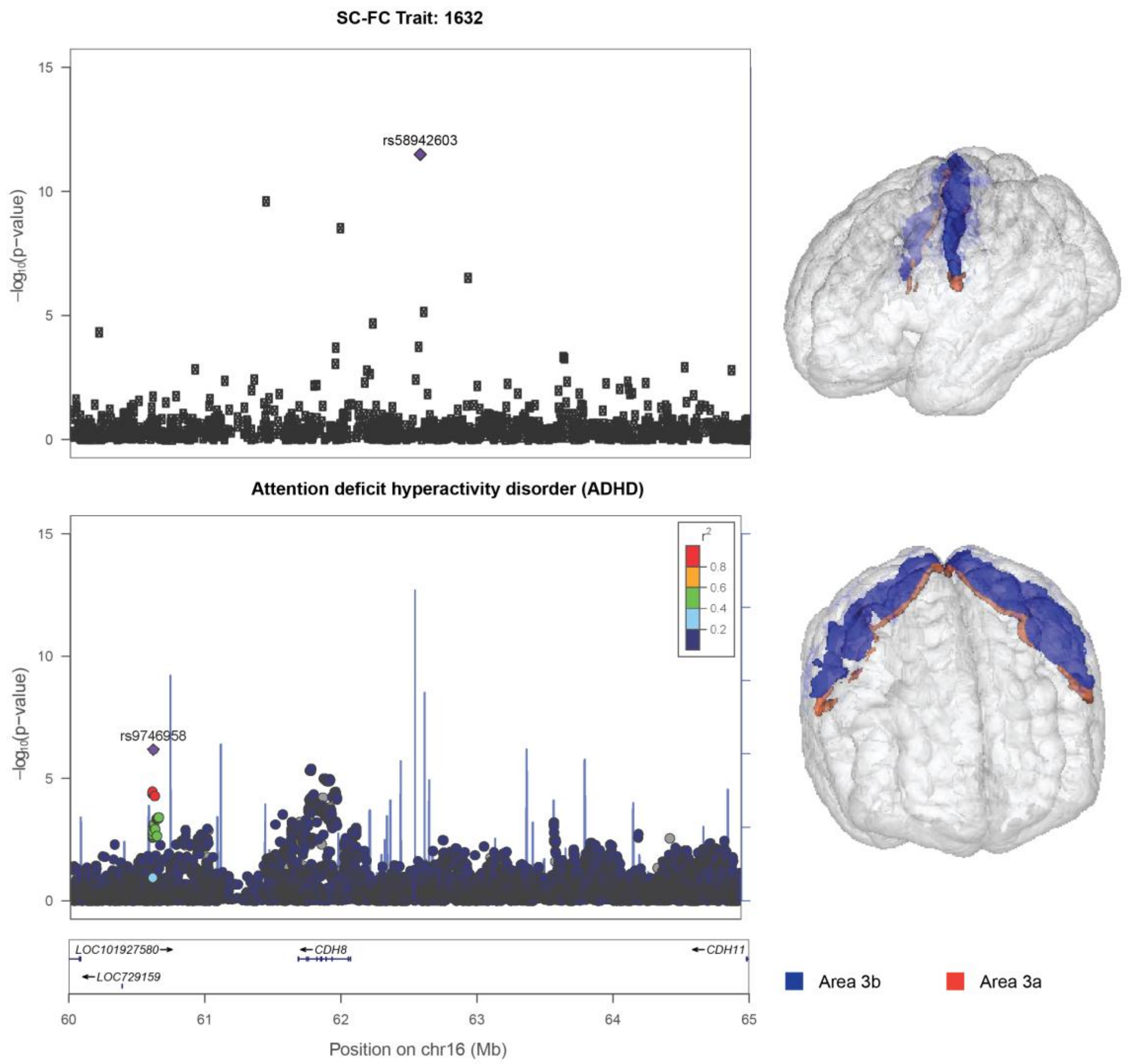

**Supplemental Fig S9.** Local colocalization between the selected SC-FC coupling trait and other brain-related complex traits/disorders in the 10p14 genomic region. Location and Glasser360 region of the displayed SC-FC coupling trait are illustrated at the bottom. Functional areas associated with the 10p14 are main in the area a32pr of Glasser360 atlas. The index variant rs498516 in the 10p14 region are reported to be associated with the left–right brain asymmetry trait with effects over anterior cingulate and medial prefrontal cortex within cingulo-opercular network.

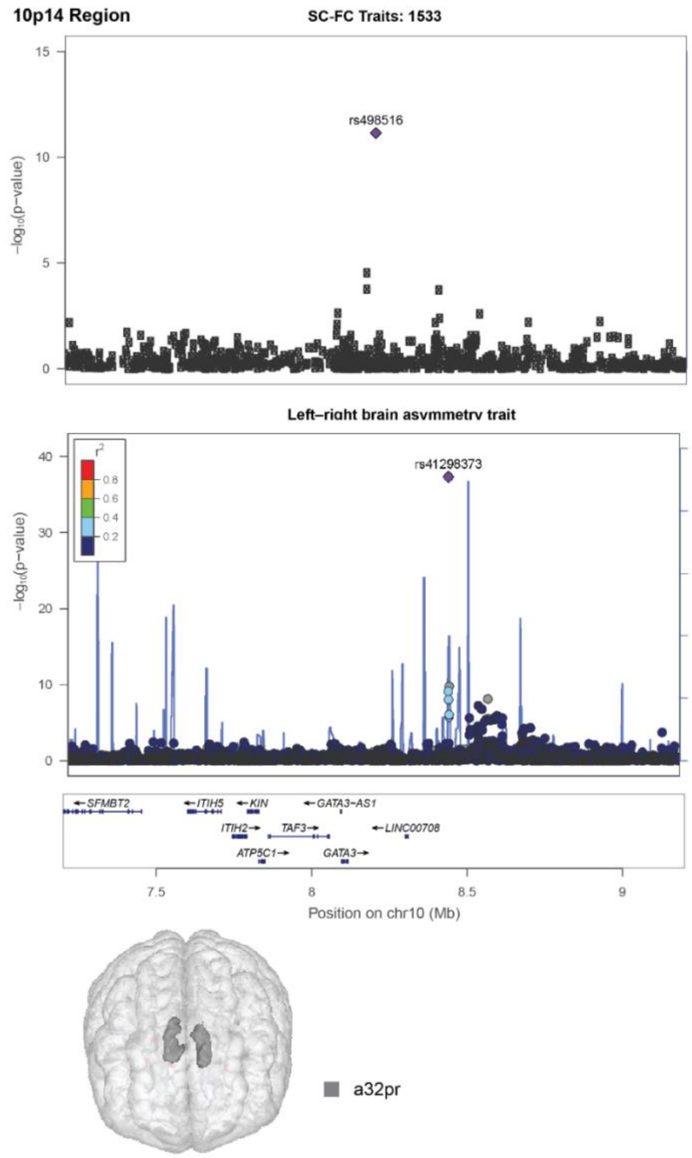

**Supplemental Fig S10.** Heritability of SC-FC Coupling for only white and non-Hispanic individuals (N = 625) and population stratification plots by first two principal components. (a) Heritability of white and non-Hispanic individuals is highly consistent with the one for all HCP individuals (r = 0.87, p = 0). (b) Scatterplot of first two principal components derived from genotype colored by different race groups. Different race groups can be stratified fairly by first two principal components. With the inclusion of first ten principal components, we believe that the varied race/ethnicity will not have much influence on the results.

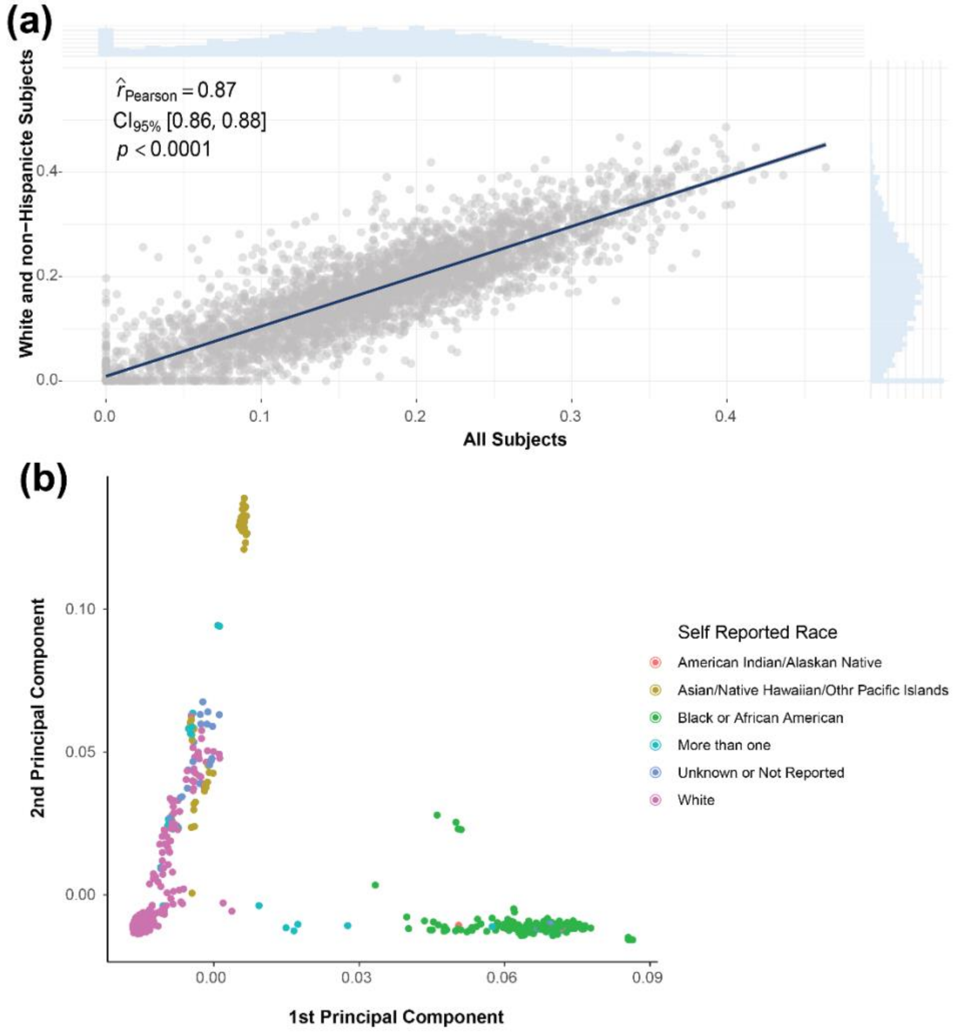

**Supplemental Fig S11.** (a) We used the same approach as that Gu et al. to construct genetic relatedness matrix and re-estimated the heritability for coupling traits. The heritability of SC–FC coupling estimated in this way are positively correlated with the original ones only using one resting-scan data (r = 0.70, p < 0.0001). (b) Heritability estimates of SC–FC coupling with first resting-scan are similar to results using second resting-scan. (c) Bland-Altmann plots of the heritability estimated using two resting scans data versus only the first resting scan data (REST1&REST2 - REST2). (d) Bland-Altmann plots of the heritability of the two resting scans (REST1- REST2). Test–retest results show good reliability and reproducibility of SC–FC coupling heritability with Pearson’s correlation of r = 0.5509, p < 0.0001.

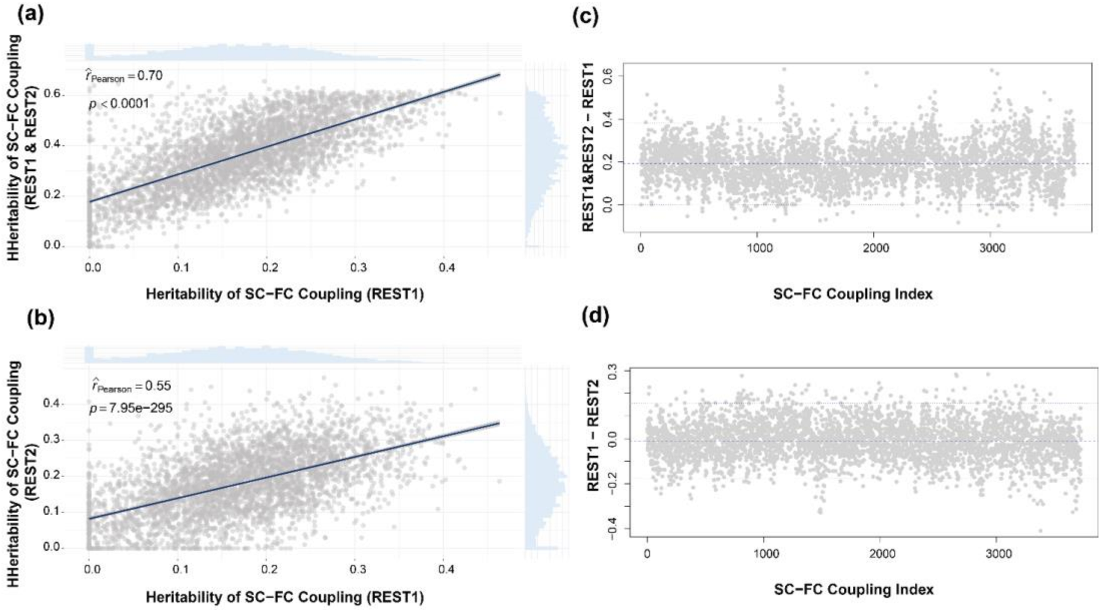

**Supplemental Fig S12.** Flowchart illustrating the selection of the HCP data for each of the analyses. We began with the S1200 HCP release including 1206 subjects with demographic and behavioral measures, of which only 1113 subjects had 3T MRI scans and 1142 subjects had genotyping data. Combined all data resources, only 899 subjects had the resting-state functional, diffusion MRI scans and genotyping data by MEGA Chip. We used these 899 in the heritability analyses, genome-wide association studies, genetic correlations and gene-level analysis in the main paper. Of them, there were 625 subjects that were white and non-Hispanic and were used in the sensitivity analysis to re-estimate heritability included in Fig. S11.

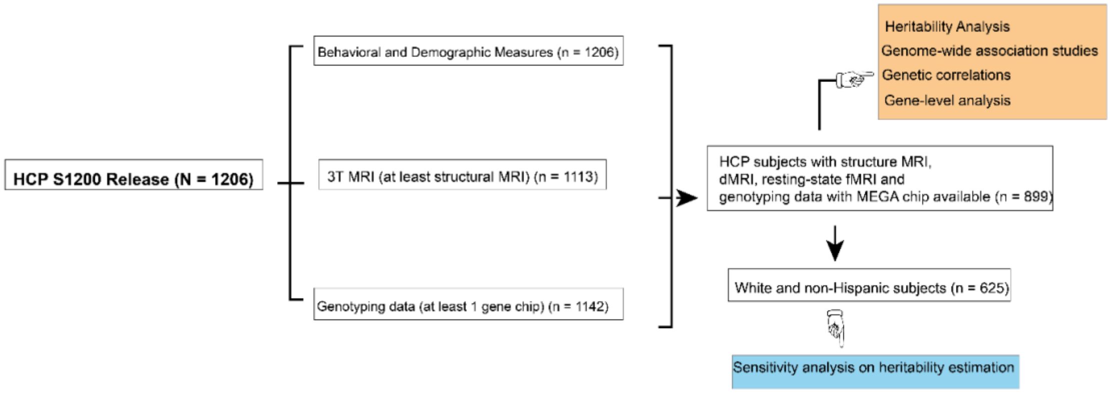

**Supplemental Fig S13.** MAGMA gene property analysis for HCP GWAS results (n=899 subjects) and 13 brain tissues. The 13 brain tissues were from GTEx v8 RNAseq database. The associations above the horizontal red dashed line are significant results ($p < 0.05$).

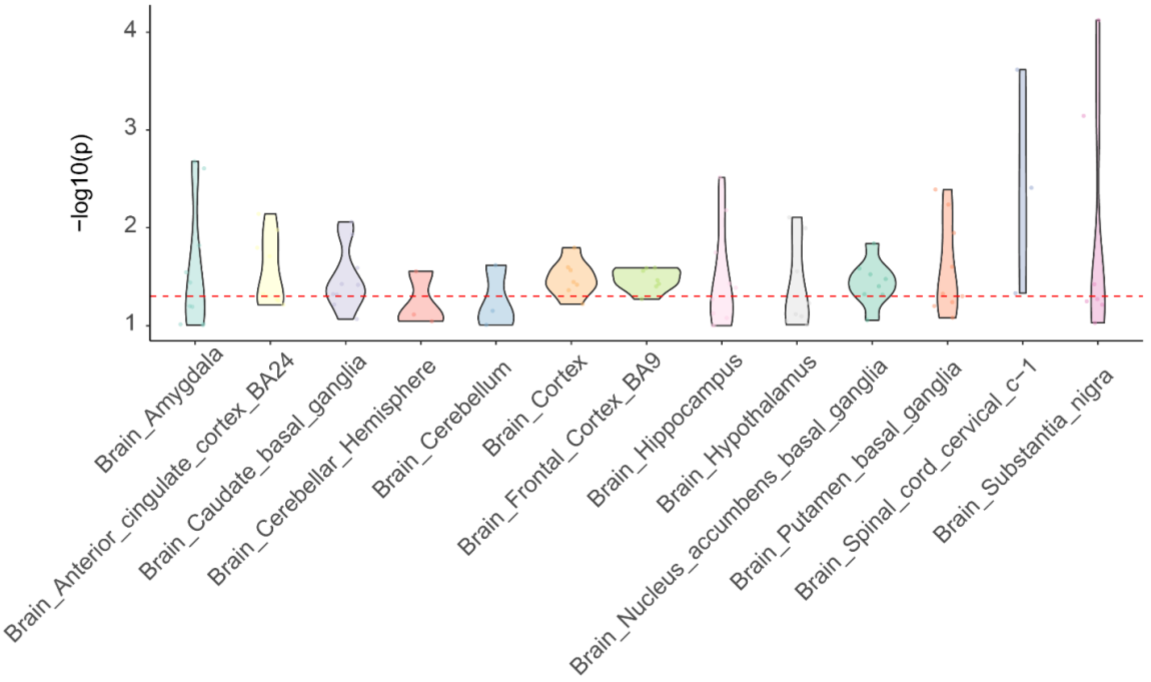

**Supplemental Fig S14.** (a) Distributions for one SC-FC coupling trait. Visually, the distribution appears to be approximately normal, with slightly skewness. (b) Manhattan plot for this trait with and without rank-based inverse normal transformation (INT). The highlighted green SNP rs77908255 is significant with rank-based inverse normal transformation at 5e-8 significance level. Other annotated SNPs are the significant ones under 1.26e-11 without transformation. (c) and (d) Q-Q plot without and with INT transformation. Both plots demonstrate a well-controlled distribution of p-values at the lower end, with some genome-wide significant findings.

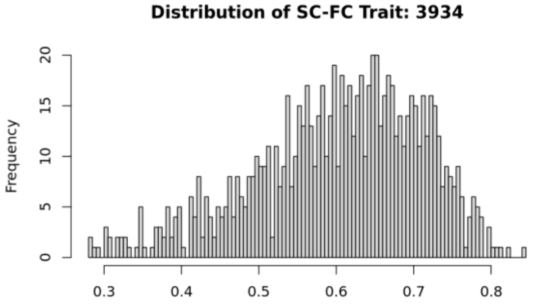


(a)

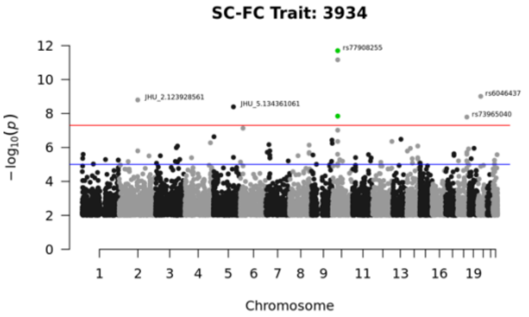


(b)

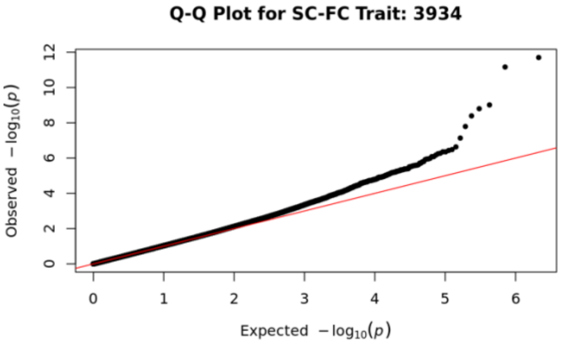


(c)

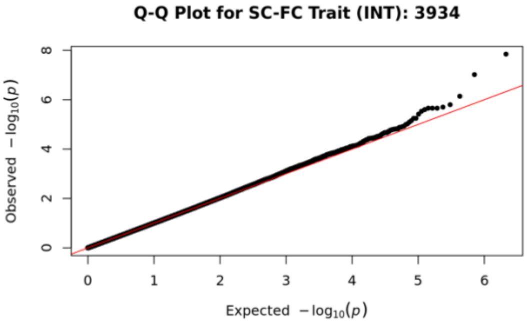


(d)

**Dataset S1 (separate file).** SNP Heritability Estimation of 3726 SC-FC coupling, FC node degree and FC node degree traits (n = 899).

**Dataset S2 (separate file).** List of significant genetic risk loci discovered for SC-FC coupling traits at $1.26 \times 10^{-11}$ significance level (n=899 subjects).

**Dataset S3 (separate file).** Validated cytoband regions in ABCD (n=1,546) with SC-FC coupling.

**Dataset S4 (separate file).** Significant ($p < 1.26 \times 10^{-11}$) variants and their correlated variants for SC-FC coupling traits that have previously been identified in GWAS of any traits listed in the GWAS catalog (version 2022-01-02)

**Dataset S5 (separate file).** Sources of the publicly available GWAS summary statistics used in this study.

**Dataset S6 (separate file).** Simulation results of the sensitivity analysis to sample size and relatedness. (a) Mean squared difference and mean standard errors of $h^2$ across different proportions of whole samples where we randomly selected a certain proportion of the samples ranging from 50% to 90% from the whole dataset. (b) Mean squared difference and mean standard errors of $h^2$ across different proportions of related samples where we adjusted the relatedness level by first only including unrelated samples (one from each family) and gradually adding 10 - 90% related samples.

**Dataset S7 (separate file).** Demographic data for subjects in the analysis. (a) Demographic data for different family structures for n = 899 samples. Age and handedness are displayed in years: mean ± SD. MZ monozygotic twins, DZ dizygotic twins. (b) Demographic data for different family structures for n = 625 white and non-Hispanic samples. Age and handedness are displayed in years: mean ± SD. MZ monozygotic twins, DZ dizygotic twins. (c) Demographic data for n = 1546 samples in ABCD study. Age is displayed in years: mean ± SD.

**Dataset S8 (separate file).** ID of 3,726 SC-FC coupling and their map onto Glasser360 atlas, 12 functional networks, 22 cortices ad 5 anatomical networks

**Dataset S9 (separate file).** Significant ($p < 2.87 \times 10^{-6}$) genes detected by MAMGA for SC-FC coupling traits that have previously been identified in GWAS of any traits listed in the GWAS catalog (version 2022-01-02).

**Dataset S10 (separate file).** MAGMA gene property analysis for HCP GWAS results (n=899 subjects) and 13 brain tissues. The 13 brain tissues were from GTEx v8 RNA-seq database. Significant tissue groupings ($p < 0.05$) are highlighted in bold

**Dataset S11 (separate file).** Significant ($P < 3.2 \times 10^{-6}$) gene sets from MAGMA gene-set analysis to prioritize the enriched biological pathways for HCP GWAS results (n=899 subjects) after Bonferroni correction.

## SI References


1. G. T. Consortium, The GTEx Consortium atlas of genetic regulatory effects across human tissues. *Science* **369**, 1318-1330 (2020).
2. p. m. h. e. Cross-Disorder Group of the Psychiatric Genomics Consortium. Electronic address, C. Cross-Disorder Group of the Psychiatric Genomics, Genomic Relationships, Novel Loci, and Pleiotropic Mechanisms across Eight Psychiatric Disorders. *Cell* **179**, 1469-1482 e1411 (2019).
3. T. B. Bigdeli *et al.*, Genome-Wide Association Studies of Schizophrenia and Bipolar Disorder in a Diverse Cohort of US Veterans. *Schizophr Bull* **47**, 517-529 (2021).
4. F. S. Goes *et al.*, Genome-wide association study of schizophrenia in Ashkenazi Jews. *Am J Med Genet B Neuropsychiatr Genet* **168**, 649-659 (2015).
5. G. Investigators, M. Investigators, S. D. Investigators, Common genetic variation and antidepressant efficacy in major depressive disorder: a meta-analysis of three genome-wide pharmacogenetic studies. *Am J Psychiatry* **170**, 207-217 (2013).
6. X. Wu *et al.*, Dysfunction of the cingulo-opercular network in first-episode medication-naive patients with major depressive disorder. *J Affect Disord* **200**, 275-283 (2016).
7. L. Dai, H. Zhou, X. Xu, Z. Zuo, Brain structural and functional changes in patients with major depressive disorder: a literature review. *PeerJ* **7**, e8170 (2019).
8. J. Martin *et al.*, A Genetic Investigation of Sex Bias in the Prevalence of Attention-Deficit/Hyperactivity Disorder. *Biol Psychiatry* **83**, 1044-1053 (2018).
9. S. Rao, A. Baranova, Y. Yao, J. Wang, F. Zhang, Genetic Relationships between Attention-Deficit/Hyperactivity Disorder, Autism Spectrum Disorder, and Intelligence. *Neuropsychobiology* **81**, 484-496 (2022).
10. N. Matoba *et al.*, Common genetic risk variants identified in the SPARK cohort support DDHD2 as a candidate risk gene for autism. *Transl Psychiatry* **10**, 265 (2020).
11. R. Karlsson Linnér *et al.*, Multivariate analysis of 1.5 million people identifies genetic associations with traits related to self-regulation and addiction. *Nat Neurosci* **24**, 1367-1376 (2021).
12. D. van der Meer *et al.*, Understanding the genetic determinants of the brain with MOSTest. *Nat Commun* **11**, 3512 (2020).
13. R. Sherva *et al.*, Genome-wide association study of rate of cognitive decline in Alzheimer's disease patients identifies novel genes and pathways. *Alzheimers Dement* **16**, 1134-1145 (2020).
14. F. R. Wendt *et al.*, Multivariate genome-wide analysis of education, socioeconomic status and brain phenome. *Nat Hum Behav* **5**, 482-496 (2021).
15. M. Liu *et al.*, Association studies of up to 1.2 million individuals yield new insights into the genetic etiology of tobacco and alcohol use. *Nat Genet* **51**, 237-244 (2019).
16. Z. Sha *et al.*, The genetic architecture of structural left-right asymmetry of the human brain. *Nat Hum Behav* **5**, 1226-1239 (2021).
17. C. A. de Leeuw, J. M. Mooij, T. Heskes, D. Posthuma, MAGMA: generalized gene-set analysis of GWAS data. *PLoS Comput Biol* **11**, e1004219 (2015).
18. A. Liberzon *et al.*, Molecular signatures database (MSigDB) 3.0. *Bioinformatics* **27**, 1739-1740 (2011).
19. A. A. Shadrin *et al.*, Vertex-wise multivariate genome-wide association study identifies 780 unique genetic loci associated with cortical morphology. *Neuroimage* **244**, 118603 (2021).
20. G. Davies *et al.*, Study of 300,486 individuals identifies 148 independent genetic loci influencing general cognitive function. *Nat Commun* **9**, 2098 (2018).
21. E. J. Brevik *et al.*, Genome-wide analyses of aggressiveness in attention-deficit hyperactivity disorder. *Am J Med Genet B Neuropsychiatr Genet* **171**, 733-747 (2016).
22. C. Autism Spectrum Disorders Working Group of The Psychiatric Genomics, Meta-analysis of GWAS of over 16,000 individuals with autism spectrum disorder highlights a novel locus at 10q24.32 and a significant overlap with schizophrenia. *Mol Autism* **8**, 21 (2017).
23. K. L. Parent *et al.*, Platform to Enable Combined Measurement of Dopamine and Neural Activity. *Anal Chem* **89**, 2790-2799 (2017).

24. N. Riva *et al.*, Unraveling gene expression profiles in peripheral motor nerve from amyotrophic lateral sclerosis patients: insights into pathogenesis. *Sci Rep* **6**, 39297 (2016).
25. S. Kury *et al.*, Rare germline heterozygous missense variants in BRCA1-associated protein 1, BAP1, cause a syndromic neurodevelopmental disorder. *Am J Hum Genet* **109**, 361-372 (2022).
26. R. T. Doty, G. J. Vanasse, C. M. Disteche, D. M. Willerford, The leukemia-associated gene Mllt1/ENL: characterization of a murine homolog and demonstration of an essential role in embryonic development. *Blood Cells Mol Dis* **28**, 407-417 (2002).
27. D. Mueller *et al.*, A role for the MLL fusion partner ENL in transcriptional elongation and chromatin modification. *Blood* **110**, 4445-4454 (2007).
28. D. Frescas, D. Guardavaccaro, F. Bassermann, R. Koyama-Nasu, M. Pagano, JHDM1B/FBXL10 is a nucleolar protein that represses transcription of ribosomal RNA genes. *Nature* **450**, 309-313 (2007).
29. J. He, E. M. Kallin, Y. Tsukada, Y. Zhang, The H3K36 demethylase Jhdm1b/Kdm2b regulates cell proliferation and senescence through p15(Ink4b). *Nat Struct Mol Biol* **15**, 1169-1175 (2008).
30. W. L. Charng *et al.*, Exome sequencing in mostly consanguineous Arab families with neurologic disease provides a high potential molecular diagnosis rate. *BMC Med Genomics* **9**, 42 (2016).
31. R. K. McHugh, R. D. Weiss, Alcohol Use Disorder and Depressive Disorders. *Alcohol Res* **40** (2019).
32. P. Zheng *et al.*, Metabolite signature for diagnosing major depressive disorder in peripheral blood mononuclear cells. *J Affect Disord* **195**, 75-81 (2016).
33. A. F. Rubio-Guerra *et al.*, Depression increases the risk for uncontrolled hypertension. *Exp Clin Cardiol* **18**, 10-12 (2013).
34. B. K. Ahmedani, E. L. Peterson, K. E. Wells, L. K. Williams, Examining the relationship between depression and asthma exacerbations in a prospective follow-up study. *Psychosom Med* **75**, 305-310 (2013).
35. K. T. Watson *et al.*, Incident Major Depressive Disorder Predicted by Three Measures of Insulin Resistance: A Dutch Cohort Study. *Am J Psychiatry* **178**, 914-920 (2021).
36. L. J. Wang *et al.*, Novel plasma metabolite markers of attention-deficit/hyperactivity disorder identified using high-performance chemical isotope labelling-based liquid chromatography-mass spectrometry. *World J Biol Psychiatry* **22**, 139-148 (2021).
37. N. Mogensen, H. Larsson, C. Lundholm, C. Almqvist, Association between childhood asthma and ADHD symptoms in adolescence--a prospective population-based twin study. *Allergy* **66**, 1224-1230 (2011).
38. V. Putz-Anderson *et al.*, A behavioral examination of workers exposed to carbon disulfide. *Neurotoxicology* **4**, 67-77 (1983).
39. E. F. Gallo, J. Posner, Moving towards causality in attention-deficit hyperactivity disorder: overview of neural and genetic mechanisms. *Lancet Psychiatry* **3**, 555-567 (2016).
40. R. Caspi *et al.*, The MetaCyc database of metabolic pathways and enzymes. *Nucleic Acids Res* **46**, D633-D639 (2018).
